# CtIP-dependent nascent RNA expression flanking DNA breaks guides the choice of DNA repair pathway

**DOI:** 10.1038/s41467-022-33027-z

**Published:** 2022-09-09

**Authors:** Daniel Gómez-Cabello, George Pappas, Diana Aguilar-Morante, Christoffel Dinant, Jiri Bartek

**Affiliations:** 1 Genome Integrity Group, Danish Cancer Society Research Center, Strandboulevarden 49, Copenhagen, DK-2100 Denmark; 2 Instituto de Biomedicina de Sevilla (IBiS), Hospital Universitario Virgen del Rocío/CSIC/Universidad de Sevilla, 41013 Seville, Spain; 3 Departamento de Genética, Facultad de Biología, Universidad de Sevilla, 41012 Seville, Spain; 4 Division of Genome Biology, Department of Medical Biochemistry and Biophysics, Science for Life Laboratory, Karolinska Institute, Scheele’s vag 2, Stockholm, 17177 Sweden

**Keywords:** Targeted therapies, Genetic mapping, Cancer genomics, Super-resolution microscopy, Mechanisms of disease

## Abstract

The RNA world is changing our views about sensing and resolution of DNA damage. Here, we develop single-molecule DNA/RNA analysis approaches to visualize how nascent RNA facilitates the repair of DNA double-strand breaks (DSBs). RNA polymerase II (RNAPII) is crucial for DSB resolution in human cells. DSB-flanking, RNAPII-generated nascent RNA forms RNA:DNA hybrids, guiding the upstream DNA repair steps towards favouring the error-free Homologous Recombination (HR) pathway over Non-Homologous End Joining. Specific RNAPII inhibitor, THZ1, impairs recruitment of essential HR proteins to DSBs, implicating nascent RNA in DNA end resection, initiation and execution of HR repair. We further propose that resection factor CtIP interacts with and helps re-activate RNAPII when paused by the RNA:DNA hybrids, collectively promoting faithful repair of chromosome breaks to maintain genomic integrity.

## Abstract (web summary)

RNA has been implicated in DNA repair. This work shows that the interplay of RNAPII-generated nascent RNA, RNA:DNA hybrids and the resection factor CtIP guide DNA double strand break repair pathway choice towards error-free homologous recombination.

## Introduction

The concept of an RNA world postulates that RNA was essential for molecular processes and biochemical reactions implicated in the origin of life on Earth^1^. To compensate for RNA instability, DNA appeared later during the evolution to better preserve genetic information, followed by fidelity mechanisms to maintain genome stability^1^. Recently, RNA has emerged as a major factor in essential mechanisms regulating gene expression^2,3^ and contributing actively to DNA repair processes^4–9^. Arguably the most cytotoxic genomic lesions are DNA double-strand breaks (DSBs), lesions repaired mainly by either of the two major pathways: non-homologous end-joining (NHEJ) and homologous recombination (HR)^10–14^. While numerous protein components of these two pathways have been discovered over time, only recently RNA has been implicated in DSB repair as well. For example, recent evidence showed that DSBs in transcriptionally active genomic regions are more prone to be repaired by HR^7,8,15^.

Interestingly, only 2-8% of the human genome gets transcribed^16^, yet it is unclear, unlikely perhaps, that HR is restricted to these genomic regions only. Hence, chromatin structure at transcriptionally active sites and the influence of diverse, relevant mechanisms, including DNA repair pathways, are currently subject to intense investigation to elucidate to what extent and how RNA impacts DSB repair. So far, such efforts generated controversial results. On the one hand, global RNA transcription is inhibited after DNA damage to avoid conflicts between repair and other DNA metabolic processes such as replication^17^. RNA involvement in DNA repair processes has also been demonstrated to contribute to genomic instability by forming RNA:DNA hybrid structures^4,18^.

On the other hand, the formation of RNA:DNA hybrids regulates DNA repair in diverse organisms^7,8,19^, exemplified by the DNA damage response RNAs (DDRNAs), necessary for DDR activation^20–22^. Furthermore, recruitment of general transcription factors to DNA damage sites was reported, and an active role for RNA polymerase II (RNAPII) in DNA repair was proposed^23^. Recently, RNA polymerase III was reported to be actively recruited to DSBs by the MRN complex and mediate RNA synthesis, promoting HR repair^24^. However, any role of RNAPII, the major mammalian RNA polymerase, in this context remains unknown. Any potential mechanistic contribution to DNA repair pathway choice could also inspire cancer treatment strategies to be combined with standard-of-care DNA damaging radio-chemotherapy.

As the repair mode is critical for genomic integrity and thereby inheritance, evolution, organismal development, and tissue homeostasis, the emerging evidence for RNA involvement raises the crucial questions of whether and how could RNA guide the choice between HR and NHEJ in DSB repair, an issue that we address in our present study.

Here, we elucidate the role played by de novo RNA synthesis by RNAPII in DSB repair via HR in human cells. We found that RNA presence during different cell cycle phases impacts the decision between the HR and NHEJ repair pathways, combined with the homologous sequences of sister chromatids. Using our single-molecule analysis approaches, we observed nascent RNA overlapping with ssDNA resection tracts generated during DNA end resection, indicating that RNAs, mainly synthesized by RNAPII, are essential to initiate DNA end resection and thereby shift the choice of DSB repair towards using the more faithful HR over NHEJ. Indeed, RNA:DNA hybrid formation is essential for resection processing and as a repair regulatory step in the HR pathway. Moreover, RNAPII inhibition, using a specific CDK7 inhibitor THZ1, impairs HR factor recruitment to DSB. We further demonstrate a previously unsuspected function of CtIP as a transcription re-activator of RNAPII paused transiently by the RNA:DNA hybrids during the early stage response to DSBs, thereby promoting DNA resection and skewing DSB repair balance towards HR.

## Results

### HR factors impact ionizing radiation-induced nascent RNA

**Fig. 1:**
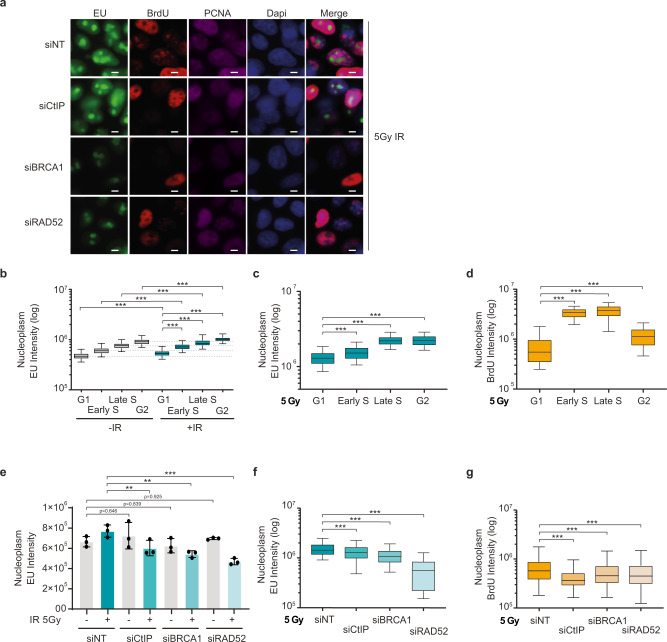
DNA resection (ssDNA) correlates with nascent RNA synthesis. **a** Representative immunofluorescence images of EU, BrdU, PCNA and DAPI staining in U2OS cells depleted for the indicated DDR factors, irradiated with 5 Gy and stained upon 30-minute labeling with EU and BrdU that started immediately after IR exposure. Scale bar: 10 μm. **b** Graph shows nucleoplasm (non-nucleolar) EU intensity in different cell cycle phases in non- and irradiated-U2OS cells. EU component was added upon DNA damage using 5 Gy in irradiated cells and labeled for 30 min in both cellular conditions. **c** Graph shows EU intensity in different cell cycle phases in control U2OS cells irradiated and labeled for 30 min as in (**a**). **d** Graph shows intensity of BrdU staining under non-denaturing conditions to visualize stretches of ssDNA, as a DNA resection marker in cell cycle phases of U2OS cells irradiated (5 Gy). **e** Bar graph represents nucleoplasm (non-nucleolar) EU intensity in cells treated by siRNAs against indicated genes in non- and irradiated cells. EU component was added upon DNA damage using 5 Gy in irradiated U2OS cells and labeled for 30 min in both cellular conditions. Mean values and ± s.e.m are represented from at least 500 cells of 3 independent experiments. Irradiated samples comparison show **p = 0.0062 (siNT vs siCtIP), **p = 0.0018 (siNT vs siBRCA1) and ***p = 0.0001 (siNT vs siRAD52) using multiple comparison with Ordinary Two-Ways ANOVA. **f** Nascent RNA synthesis in U2OS cells depleted for the indicated DDR proteins, labeled with EU for 30 min starting after irradiation with 5 Gy. **g** Quantification of BrdU staining under non-denaturing conditions to mark ssDNA as a DNA resection marker in U2OS cells depleted for CtIP, BRCA1 and RAD52, respectively, and stained after 30 min BrdU labeling started after exposure to 5 Gy. Statistical data at (**b**–**d**) and (**f**, **g**) showing mean data from 3 independent experiments. Bar plots show the median (center), 25–75 percentile (box), and 5–95 percentile (whisker) from at least 500 cells. *P* values were calculated using multiple comparison with Ordinary One-Way ANOVA. ***p < 0.001. Source data are provided as a Source data file.

To study the relationship between nascent RNA and DNA repair, we analyzed nascent RNA expression in different cell cycle phases using a modified nucleotide, 5-ethynyl uridine (EU)^25^, in human U2OS osteosarcoma cells. We focused on the time period early after irradiation since that is the time critical for the cell’s choice to repair DSBs via either NHEJ or HR. Hence our experiments were designed to analyze particularly the initial 30 min post-radiation exposure (Fig.1a). In control, proliferating and non-irradiated cells, global RNA transcription in the nucleoplasm increased during the cell cycle, reaching maximum levels in S and G2 phases (Fig.1b). The overall cell cycle pattern of nascent RNA transcription was similar in irradiated cells, however reaching higher levels in each of the examined cell cycle phases (G1, early S, late S and G2, respectively), compared to non-irradiated controls (Fig.1a–c). Such RNA increase was transient, returning to pre-irradiation levels by 60 min after radiation exposure (Supplementary Fig. 1a). While HR repair is more active in S and G2 phases due to availability of sister chromatids, we wondered whether DNA resection, as a critical upstream step in HR, follows a similar cell cycle distribution pattern related to nascent RNA transcription. Visualization of ssDNA using BrdU detection under non-denaturing conditions by immunofluorescence showed that following irradiation with 5 Gy, DNA end resection was low in G1, reached maximum levels in S, followed by a decrease in G2, albeit to a level that was still clearly higher compared to G1 (Fig. 1c, d). Although these cell cycle-related trends of EU and BrdU labeling after radiation were not entirely parallel due to the partial difference in G2, the profiles of both nascent RNA transcription and DNA end resection shared the lowest values in G1 compared to S and G2 phases, raising the possibility of a functional link between these two processes. The presence of CtIP and BRCA1 proteins promote DNA end resection at DSBs^26^. To determine any potential role of these proteins in de novo RNA transcripts formation after DNA damage, we compared nascent RNA in CtIP- and BRCA1-depleted cells, respectively, with mock-depleted controls, under both non-irradiated and irradiated conditions (Fig. 1e and Supplementary Fig.1b–d). For such depletion experiments, we used previously published, validated siRNA sequences against these two genes^25–27^. Whereas CtIP- and BRCA1-depleted non-irradiated cells showed unaltered global RNA transcription at 30 min and 60 min (Fig. 1e and Supplementary Fig. 1b), the nascent RNA expression was decreased in the irradiated cells depleted of either CtIP or BRCA1 (Fig. 1e, f).

Furthermore, reduced RNA transcription after irradiation was observed to different extents in various cell cycle phases in cells which were pre-depleted of HR factors CtIP, BRCA1, or RAD52 (Fig. 1f and Supplementary Fig. 2a). These results are consistent with the recognized role of BRCA1 in transcriptional regulation of RNAPII, along with transcriptional activators such as p300/CBP^27^. CtIP depletion negatively affected nascent RNA predominantly in the late S and G2, having less impact in the early S phase (Supplementary Fig. 2a), where CtIP levels are still low. As expected, both BRCA1 and CtIP knockdown reduced ssDNA generation by resection in all cell cycle phases (Fig. 1g and Supplementary Fig. 2b), while the CtIP-depleted cells showed a more pronounced resection defect in the G2 phase after radiation, again coinciding with less nascent RNA (Supplementary Fig. 2a, b).

Recently, it has been reported that RAD52 recruits BRCA1 in transcription-associated HR repair^8^. Strikingly, the knockdown of RAD52 impaired the nascent RNA synthesis and DNA resection at IR-induced DSBs (Fig. 1e–g and Supplementary Fig. 2a, b) but not significantly in non-irradiated cells (Fig. 1e and Supplementary Fig. 1b). Such nascent RNA and resection deficiencies associated with RAD52 depletion occur in all cell cycle phases upon irradiation (Supplementary Fig. 2a, b), indicating that both defects occur in a cell cycle-independent manner. This, in turn, suggests that RAD52 plays a role in RNA transcription in response to DNA damage, at least during the first 60 min upon irradiation. Additionally, depletion of 53BP1, known for its role in promoting NHEJ, did not alter nascent RNA in either non- or irradiated-U2OS cells at either 30 or 60 min after irradiation (Supplementary Fig.2 c, d). Taken together, these results indicate that increased nascent RNA transcription and more active DNA resection in response to IR-generated DSBs are higher in S and G2 phases compared with G1, suggesting that these processes may be functionally linked. Relevance of such emerging transcription-resection interplay for DSB repair is further supported by the observed transcriptional decrease in cells depleted of HR factors CtIP and BRCA1, in contrast to no such effect upon knockdown of the NHEJ-associated factor 53BP1.

### Nascent RNAs generated after DNA damage colocalize with DNA resection tracts

**Fig. 2:**
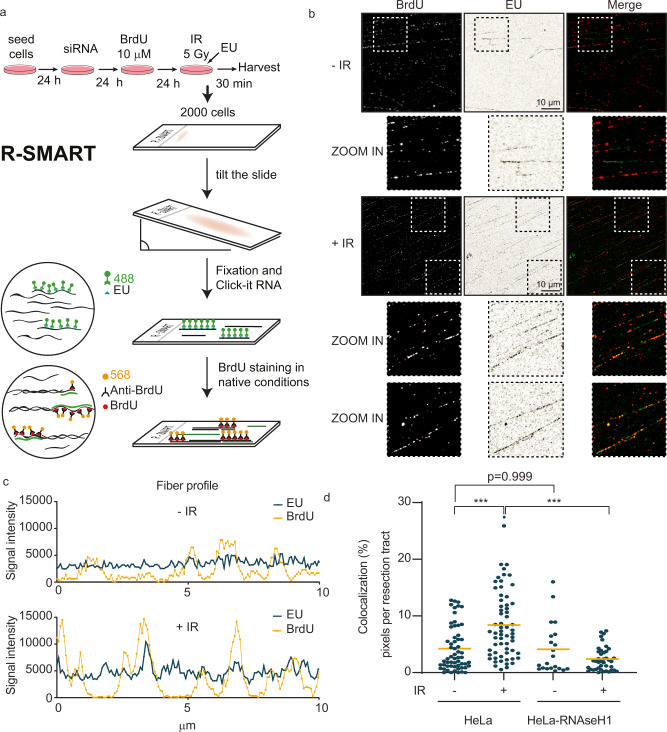
Nascent RNAs colocalize with DNA resection tracts in irradiated HeLa cells. **a** Schematic representation of the developed R-SMART technique. **b** Representative images of DNA resection tracts (black background) and nascent RNAs (white background) upon 5 Gy irradiation, using non-denaturing conditions for BrdU staining and EU labeling, respectively. *n* = 3 biologically independent experiments. Scale: 10 μm. **c** Representative quantification of fiber profiles for EU and BrdU intensities from non- and irradiated HeLa cells. *n* = 3 biologically independent experiments. **d** Dot graph shows percentage of BrdU and EU signal colocalization on resection tracts generated in non- and 5 Gy-irradiated HeLa and HeLa-RNAseH1 cells. At least n = 60 fields from 3 independent experiments were quantified. P values were calculated using multiple comparison with Ordinary One-Way ANOVA. ***p < 0.0001. Source data are provided as a Source data file.

Next, we investigated whether the new RNA synthesis after DNA damage is required to facilitate DSB repair by HR. For this purpose, we developed a technique to simultaneously observe nascent RNA and DNA resection tracts based on the principle of the nucleic acid (NA) fibers approach. Our technique, called R-SMART (RNA-SMART) to acknowledge a modification of the SMART assay^26^, requires incubation with the RNA precursor 5-ethynyl uridine (EU) upon IR. To perform this analysis, HeLa cells were treated with BrdU for 24 h, followed by a 30 min EU pulse immediately after IR exposure, before stretching the NA fibers (Fig. 2a). The main objective of this technique is to quantify nascent RNA transcripts (through detection of the pulse-incorporated EU) only in DNA resection tracts. We measure the colocalization of EU labeling and staining for BrdU under native conditions, allowing BrdU visualization in ssDNA, thus marking the DNA resection tracks (Fig. 2a, b). We observed an increased BrdU signal in fibers from cells exposed to 1 Gy and 5 Gy of ionizing radiation (Supplementary Fig. 3a). Using the colocalization R-SMART technique, we detected an increased number of ssDNA fibers in irradiated cells, using native conditions for anti-BrdU staining to visualize activation of DNA resection (Fig. 2b and Supplementary Fig. 3b). Hence, the R-SMART technique can differentiate the levels of ssDNA in cell responses to different radiation doses (Supplementary Fig. 3a) and can detect a deficiency in HR factors upon irradiation (Supplementary Fig. 3b). Notably, profile analysis of BrdU-marked ssDNA resection tracts determined on the DNA fibers showed overlapping EU-labeled nascent RNA peaks, with colocalization of both accentuated in IR-treated cells (Fig. 2b–d). To better determine the impact of irradiation, we made a quantitative comparison of the overlap between nascent RNA and ssDNA in irradiated *versus* control cells. A more pronounced EU staining above the DNA resection tracts, seen in response to IR, suggested that nascent RNAs are linked with ssDNA generation (DNA end resection), the critical step of HR repair (Fig. 2c, d).

Based on these results, we would predict that the newly generated RNA at DSB regions would have high affinity and complementarity for the ssDNA stretches generated by resection during HR, thus likely creating DNA:RNA hybrid structures susceptible to enzymatic degradation by RNAseH1. This prediction was tested and confirmed, taking advantage of a validated cell line HeLa-RNAseH1 with stable expression of ectopic RNAaseH1, a model useful for assessing cellular roles of RNA:DNA hybrids^28,29^. RNaseH1 expression reduced the extent of post-IR interaction between ssDNA and nascent RNA (BrdU colocalization with EU), consistent with a scenario that ssDNA tracts may indeed be covered or protected in some regions from degradation by nascent complementary RNA molecules (Fig. 2d). Altogether, these results support the notion that de novo RNA synthesis after DSB generation is closely linked to DNA resection during the early stage of DSB repair by HR.

### DNA damage stimulates RNA:DNA hybrid formation on DNA resection tracts

**Fig. 3:**
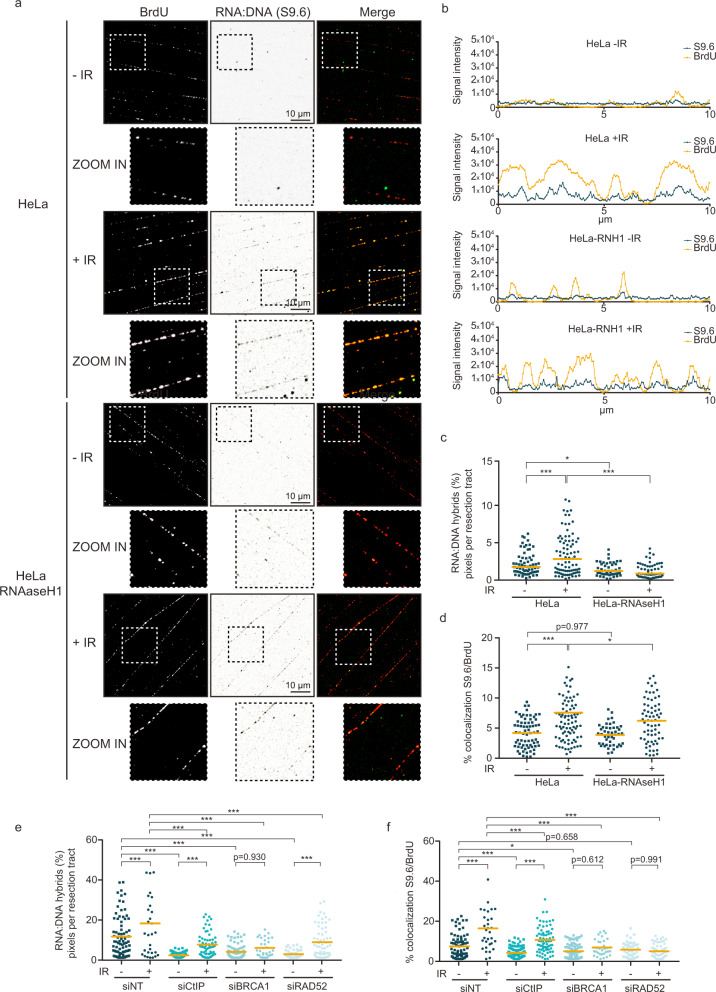
DNA damage generates high frequency of RNA:DNA hybrids on resection tracts. **a** Representative images showing ssDNA (resection tracts) and RNA:DNA hybrids upon non- and 5 Gy-irradiated HeLa cells: non-denaturing BrdU staining and S9.6 staining, respectively. *n* = 3 biologically independent experiments. Scale 10μM **b** Representative quantifications of fiber profiles for DNA resected track (BrdU) and RNA:DNA hybrids (S9.6) staining intensities from non- and irradiated HeLa and HeLa-RNAseH1 cells. **c** Dot graph shows percentages of RNA:DNA hybrids (S9.6) staining signal on resection tracts generated in non- and 5 Gy-irradiated HeLa and HeLa-RNAseH1 cells. *P* values were calculated using multiple comparison with Ordinary One-Way ANOVA. ***p = 0.0006 (HeLa −IR vs HeLa +IR), *p = 0.047 (HeLa −IR vs HeLa-RNAseH1 −IR) and ***p < 0.0001 (HeLa +IR vs HeLa-RNAseH1 +IR). **d** Dot graph shows mean of percentages of RNA:DNA hybrids (S9.6) and ssDNA (BrdU) signal colocalization on resection tracts generated in non- and 5 Gy-irradiated HeLa and HeLa-RNAseH1 cells. *P* values were calculated using multiple comparison with Ordinary One-Way ANOVA. *p = 0.044 (HeLa +IR vs HeLa-RNAseH1 +IR) and ***p < 0.0001 (HeLa −IR vs HeLa +IR). **e** HeLa cells transfected with siRNA against CtIP, BRCA1, RAD52, and NT (Non-Target) for 48 h, were assessed for RNA:DNA hybrids (S9.6) quantification by RL-SMART as in (**c**). *P* value were calculated using multiple comparison with Ordinary One-Way ANOVA. ***p < 0.001 **f** HeLa cells transfected with siRNA against CtIP, BRCA1, RAD52, and NT (Non-Target) for 48 h were assessed for RNA:DNA hybrids (S9.6) and ssDNA (BrdU) and quantify the percentage of colocalization as (**d**). *p = 0.048, ***p < 0.001 using multiple comparison with Ordinary One-Way ANOVA. **b**–**f** At least 30 fields from n = 3 independent experiments were quantified. Source data are provided as a Source data file.

As RNAseH1 impacted the new RNA synthesis over ssDNA resection tracts in DNA-damaged cells, we next investigated whether such resection-associated RNA:DNA hybrids were detectable using the commonly employed S9.6 antibody^4^. We took advantage of another optimized nucleic acid fibers-based technique, developed by us, called RL-SMART to visualize S9.6 staining in non-denatured nucleic acid fibers labeled and co-stained by antibody to BrdU (Fig. 3a, b and Supplementary Fig. 4a). Using this technique, we detected a radiation-induced increase of RNA:DNA hybrids in nucleic acid fibers from cells exposed to 1 and 5 Gy doses (Supplementary Fig. 4b), with up to 49,1% more RNA:DNA hybrids found in the irradiated samples (Fig. 3c, and Supplementary Fig. 5a). Specific controls for this RL-SMART technique included RNAseA and RNAseH treatments to remove RNA:DNA hybrids, thereby validating the data obtained using the S9.6 antibody (Supplementary Fig. 5b). An additional control to validate the RL-SMART approach included treatment of cells with Pladionalide B, a powerful RNA splicing inhibitor that provokes R-loop accumulation^30^, further documenting that RNA:DNA hybrids, and not other structures are detected by the RL-SMART technique (Supplementary Fig. 5c–e).

While only a fraction of RNA:DNA hybrids coincided with BrdU staining in the same track, the incidence of RNA:DNA staining colocalized with ssDNA tracts increased by 85% in irradiated cells (Fig. 3a, b). In parallel control cells, such RNA:DNA hybrid signal over ssDNA resection tracts was reduced when cells expressed ectopic RNAseH1 (Fig. 3a, b). RL-SMART profile analysis revealed that the RNA:DNA hybrid-staining peaks coincided with higher DNA resection staining peaks, indicating that the RNA:DNA hybrids indeed localize to the resection tracts generated upon irradiation (Fig. 3a, b). As expected, RNAseH1 overexpression in HeLa cells abolished RNA:DNA hybrids on the resection tracts (Fig. 3a, b). Quantification of RNA:DNA hybrids showed a 48% and 52% reduction of RNA:DNA hybrids seen in non- and IR-exposed HeLa-RNAseH1 cells, respectively, compared with HeLa control cells without ectopic RNAseH1 (Fig. 3c, d). Some residual RNA:DNA hybrids remained present despite RNAseH1 treatment, suggesting that some RNA:DNA hybrids may not be entirely accessible and could thus be protected against degradation during DNA resection at DSBs and/or require other mechanisms to be resolved.

Next, we studied the role of HR factors in RNA:DNA hybrid formation during DNA resection using the RL-SMART technique. Consistently with the above results, when HeLa cells were depleted for CtIP, BRCA1 and RAD52 proteins, respectively, we observed over 50% decreased RNA:DNA hybrid formation in all cases (Fig. 3e, f). These results most likely reflect the decreased formation of nascent RNA during the DNA repair by HR (Fig. 3e, f). The amount of RNA:DNA hybrids was reduced in both, irradiated and non-irradiated cells, the latter result likely reflecting repair of endogenous DNA damage, possibly caused by cancer-associated endogenous replication stress and the ensuing DSBs^25^, the repair of which is also affected by depletion of these DDR factors. Interestingly, irradiated CtIP-depleted cells still generated detectable RNA:DNA hybrids, albeit reduced by 61,9% in comparison with the irradiated CtIP-proficient controls (Fig. 3e, f). Cells depleted of RAD52 also showed a reduction in RNA:DNA structures, the incidence of which can increase after irradiation (Fig. 3e). In the latter scenario, colocalization of RNA:DNA hybrids with DNA resection tracts did not increase (Fig. 3f), suggesting that RAD52 may contribute to possible R-loop formation close to the RNAPII and DNA resection machineries that involve ssDNA displaced upon RNA:DNA hybrid formation. BRCA1 protein was also required for RNA:DNA hybrid formation after DNA damage, in terms of both the overall extent and colocalization with DNA resection tracts (Fig. 3e, f). Taken together, these results indicate that new transcript RNAs generate RNA:DNA hybrids using 3′−5′ssDNA, thereby providing an additional level of regulation over DNA resection, suggesting a potential uncharacterized role of CtIP and BRCA1 in DNA repair pathway choice.

### RNAPII inhibition impairs the recruitment of HR proteins

**Fig. 4:**
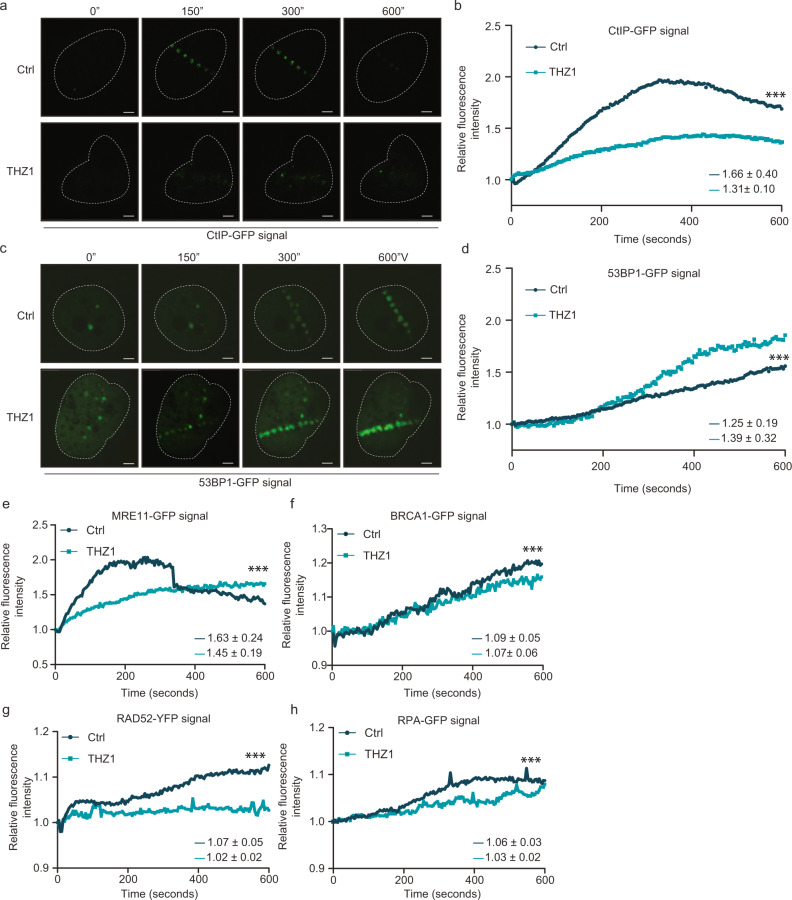
Inhibition of RNAPII impairs DDR protein recruitment to DSBs. **a** Representative time-lapse images of CtIP-GFP recruitment to DNA damage in RNAPII -inhibited (THZ1, 1 µM during 2 h) and DMSO as control during 600 s post microlaser irradiation in U2OS cells. *n* = 3 biologically independent experiments. Scale bar: 1 μm **b** Effect of THZ1 treatment (1 µM during 2 h) on the kinetics of CtIP-GFP recruitment to DNA damage by measuring relative fluorescence intensity of CtIP-GFP during 600 s post microlaser irradiation in U2OS cells. The analysis represents the average on *n* = 30 (Ctrl) and *n* = 15 (THZ1) nuclei from 3 biologically independent experiments. *p* values were calculated using two-tailed paired *t* test. ***p < 0.0001 **c** Representative images of 53BP1-GFP recruitment to DNA damage after THZ1 treatment (1 µM during 2 h), assessed as in (**a**). *n* = 3 biologically independent experiments. Scale bar: 1 μm **d** Kinetics of 53BP1-GFP intensity in U2OS cells treated with DMSO or THZ1 (1 µM during 2 h) prior microlaser irradiation, measured as in (**b**). The analysis represents the average on *n* = 17 (Ctrl) and *n* = 18 (THZ1) nuclei from 3 biologically independent experiments. *p* values were calculated using two-tailed paired *t* test. ***p < 0.0001. **e**–**h** Kinetics of recruitment to laser-induced DNA damage, for DDR factors MRE11 (**e**), BRCA1 (**f**), RAD52 (**g**) and RPA (**h**) treated or not with RNAPII inhibitor (THZ1, 1 µM during 2 h) prior microlaser irradiation, and measured as in (**b**). The analysis represents the average of nuclei from 3 biologically independent experiments. MRE11, *n* = 31 (Ctrl) and *n* = 29 (THZ1); BRCA1, *n* = 18 (Ctrl) and *n* = 14 (THZ1), RAD52, *n* = 26 (Ctrl) and *n* = 8 (THZ1), RPA, *n* = 17 (Ctrl) and *n* = 16 (THZ1). *p* values were calculated using two-tailed *t* test in all graphs. ***p < 0.0001. **b**, **d, e**–**h**, two-tailed paired *t* test was analyzed for all kinetics. ***p < 0.001. In the graphs the mean of mobile fractions and the ±SD (bottom right) are shown for each sample. Source data are provided as a Source data file.

Given our results so far and the fact that RNA polymerase II (RNAPII) gets recruited to DSBs^21,23^, we hypothesized that RNAPII accumulation and newly produced RNA could be involved in choosing between the two main DSB repair pathways. To investigate this hypothesis, we used a THZ1 compound, a potent inhibitor of CDK7, indeed of RNAPII-mediated RNA transcription^31^, to assess the impact of RNA synthesis inhibition on recruitment of DDR proteins to DSB. We monitored phosphorylation of Serine 5 on CTD repeats of RNAPII by CDK7, a modification which is essential for transcription initiation. We observed specific inhibition of phosphorylation of this residue upon THZ1 treatment (Supplementary Fig. 6a) and as a consequence nascent RNA decrease (Supplementary Fig. 6b). Next, we analyzed the recruitment of the main DSB repair factors tagged with green fluorescence protein (GFP) to microlaser irradiation-created, DSB-rich DNA damage tracts^32^. Given that CtIP is a key protein for HR-mediated DSB repair, playing a major role in the activation of DNA resection^33–35^, and our present data showing the impact of CtIP on radiation-induced RNA:DNA hybrid formation, we evaluated CtIP-GFP dynamics on DNA damage sites in THZ1-treated cells (Fig. 4a, b). THZ1-mediated RNAPII inhibition reduced the CtIP-GFP recruitment to DSBs during the initial 10 min after microirradiation (see also the supplementary movie 1). These results suggested that active RNAPII is required for the initiation of CtIP-dependent DNA resection. It is well known that HR deficiency leads to DSB repair shift towards NHEJ-mediated repair^13,14^. Notably, studying 53BP1-GFP kinetics in the presence of RNAPII inhibitor, we found that 53BP1 recruitment was more abundant and faster than in control vehicle-treated cells (Fig. 4c, d), suggesting that DNA resection deficiency due to RNAPII inhibition favors the NHEJ pathway. Whereas the DSB repair shift towards NHEJ occurs around 4 min (240 sec) after irradiation, the impairment of CtIP recruitment is apparent already during the first 2 min after laser microirradiation. When other GFP-tagged DDR proteins were evaluated, we found that MRE11, another key resection protein, also displayed an acutely impaired recruitment to DSBs under THZ1 treatment (Fig. 4e). These data suggest that CtIP and MRE11, as key DNA resection factors, require RNAPII activation at DSB sites to promote the DNA resection machinery assembly and thereby engagement of the HR repair pathway. BRCA1 and RPA recruitment were also reduced upon RNAPII inhibition but slightly later than CtIP and MRE11, suggesting that these factors help DNA resection processing at a later step rather than at the initiation. Indeed, BRCA1 is essential to regulate resection speed and RPA protects ssDNA after resection^36^ (Fig. 4f, h). Furthermore, recruitment of RAD52, a protein shown to promote transcription-coupled HR repair, to the DNA repair complex was also impaired under THZ1 treatment (Fig. 4g). Taken together, our data show that RNAPII activity supports efficient recruitment of HR repair factors, and inhibition of RNAPII favors NHEJ repair through preferential recruitment of 53BP1 early in the chromatin response to DSBs.

### Functional RNAPII shifts the DSB repair balance towards HR

**Fig. 5:**
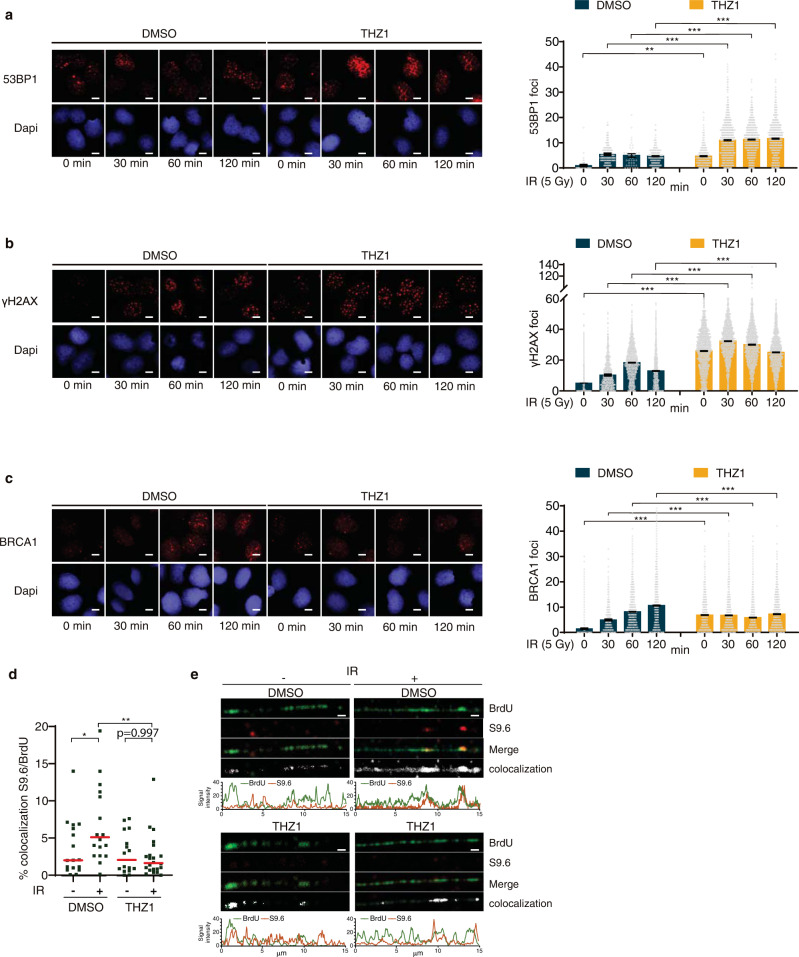
Defective recruitment of HR factors after RNAPII inhibition favors NHEJ repair. **a** Representative images of 53BP1 foci upon 5 Gy irradiation in U2OS cells treated with DMSO or THZ1 (1 µM during 2 h), (left). Scatter dot blot for 53BP1 foci quantification at 0, 30, 60, and 120 min after irradiation (right). **b** Same as in (**a**), but measuring γH2AX foci. **c** Same as in (**a**), but measuring BRCA1 foci. **a**–**c** At least *n* = 500 cells examined over 3 independent experiments were quantified. Data are presented as mean values ± s.e.m. *p* values were calculated using multiple comparison with Ordinary One-Way ANOVA. **p = 0.002 and ***p < 0.0001 Scale bar: 10 μm. **d** Dot graph of RL-SMART assay shows mean of percentages of RNA:DNA hybrids (S9.6) and ssDNA (BrdU) signal colocalization on resection tracts generated in non- and 5 Gy-irradiated HeLa cells. p value were calculated using multiple comparison with Ordinary One-Way ANOVA. *p = 0.036 and **p = 0.002. At least *n* = 15 fields examined over 3 independent experiments were quantified. **e** Representative quantifications of fiber profiles for DNA resected track (BrdU) and RNA:DNA hybrids (S9.6) staining intensities from non- and irradiated HeLa cells treated with RNAPII inhibitor, THZ1 (1 μM 2 h). *N* = 3 independent experiments. Scale bar: 1 μm. Source data are provided as a Source data file.

Next, we wished to further extend the concept emerging from our present results, namely that new RNAPII-mediated transcription at DSB sites is required to direct the cell’s choice of DSB repair process by promoting accrual of essential HR factors. First, we demonstrated that recruitment of RPA, which is required to protect ssDNA stretches created during DNA end resection, diminished at DSBs during the first 10 min (600 s) after microirradiation (Fig. 4h). Next, we examined whether cells exposed to ionizing radiation and treated with THZ1 alter their choice of DNA repair mode in the longer term. We monitored S4/S8 phosphorylated RPA foci formation as a DNA resection marker until two hours post-irradiation. Indeed, THZ1-treated cells became defective in their ability to form foci of phosphorylated RPA (Supplementary Fig. 7a). When testing the kinetics of IR-induced foci formation by other DDR factors in THZ1-treated cells, we observed enhanced accumulation of 53BP1 to DSB-flanking chromatin, associated with an increase of nuclear foci formation of 53BP1 until 2 h after irradiation under THZ1 treatment (Fig. 5a). 53BP1 foci accumulation in THZ1-treated cells was almost 5-fold increased during 1 h even without any exogenous genotoxic insult. This enhanced DNA damage load suggests that under THZ1-mediated RNAPII inhibition conditions, endogenously arising DNA lesions may be preferentially repaired by the error-prone NHEJ pathway and, possibly more important, that the trapped inhibited RNAPII complexes increase the frequency of damage-prone replication-transcription conflicts.

Furthermore, we tested RIF1, as a 53BP1 interactor when phosphorylated by ATM to promote NHEJ, in THZ1-treated cells and showed that RNAPII inhibition resulted in increased RIF1 foci formation upon IR as well (Supplementary Fig. 7b).

The THZ1-inhibited cells showed more unresolved DNA damage associated with enhanced DNA damage marker formation before and after irradiation (Fig. 5a–c), suggesting that RNAPII inhibition fuels the accumulation of endogenous DNA damage. Furthermore, foci formation by BRCA1 and RAD51, factors involved in early and late HR steps, respectively, was impaired at multiple time points post-irradiation in the THZ1-treated cells (Fig. 5c and Supplementary Fig. 7c). Next, to corroborate the notion that transcription initiation is essential to promote HR, we treated cells with Triptolide (TRP), another inhibitor of RNAPII initiation step through TFIIH. Consistent with the data obtained with THZ1, treatment with TRP led to impaired radiation-induced foci formation by phosphorylated RPA and BRCA1, contrary to 53BP1 foci formation that remained unaffected (Supplementary Fig. 8a, b). The observed lack of enhanced 53BP1 foci likely reflects that fact that TRP inhibits transcription by inducing proteasome-mediated degradation of RNAPII, thereby avoiding the increase of transcription-replication conflicts that occured in response to THZ1 treatment.

Finally, we investigated the RNA:DNA hybrid formation in DNA resection tracks upon irradiation under RNAPII inhibition by THZ1 using the RL-SMART assay. Consistent with both nascent RNA and HR recruitment impairment after RNAPII inhibition, we observed a 70% reduction of colocalization between BrdU and S9.6 staining in irradiated in THZ1-treated cells (Fig. 5d). Supporting our previous data, we observed specific regions inside DNA resection tracks with high colocalization peaks after irradiation, the extent of which was reduced in THZ1-treated cells (Fig. 5e). Again, TRP treatment led to results similar to those obtained with THZ1, showing decreased RNA:DNA hybrid formation in post-IR DNA resection tracks (Supplementary Fig. 8c). Thus, we propose that nascent RNA synthesis is an essential step to promote DSB repair via HR and that formation of RNA:DNA hybrids at DSBs provides an intermediate structure required to carry out the early phase of HR.

### CtIP is required to re-initiate transcription transiently paused after DNA damage

**Fig. 6:**
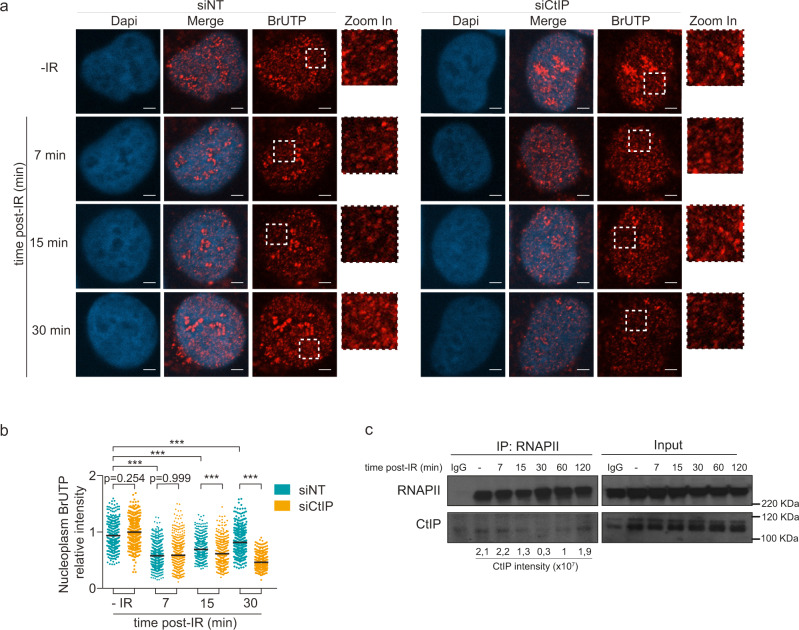
DNA resection deficiency prevents transcription re-start after DNA damage. **a** Representative images of de novo transcription based on BrUTP incorporation assay at 7, 15 and 30 min post-IR (5 Gy) in CtIP-depleted U2OS cells. Zoom in pictures are shown. *n* = 3 biologically independent experiments. Scale bar: 1 μm. **b** Dot graph shows nucleoplasm (non-nucleolar) quantification of BrUTP incorporation under the experimental conditions cued in (**a**). At least *n* = 200 cells examined over 3 independent experiments were quantified. Data are represented as mean value. *P* value were calculated using multiple comparison with Ordinary One-Way ANOVA. ***p < 0.0001. **c** RNAPII immunoprecipitation from extracts of non- and irradiated U2OS cells at 7, 15, 30, 60 and 120 min post-IR (5 Gy). Immunoblot detection of CtIP and RNAPII proteins in immunoprecipitated and input samples, as indicated. Quantification of CtIP co-immunoprecipitated with RNAPII is shown below. *N* = 3 independent experiments. Source data are provided as a Source data file.

Apart from indicating that the observed DSB-associated transcription shifts the DSB repair pathway choice towards HR, our results also suggested that the DNA resection factors could be promoting transcription to facilitate 5′−3′ single-strand DNA degradation. Indeed, while CtIP and BRCA1 are critical for DNA resection during HR repair, these factors are also known to function as transcription factors^37,38^, albeit the molecular basis for the latter role and how it may be coordinated with the DNA resection role during DSB repair, are not fully understood. Thus, we hypothesized that CtIP could regulate transcription at DSBs, thereby allowing other DNA resection proteins to be recruited. To address this possibility, we first tested de novo RNA transcription in CtIP-deficient cells before and at early time points after irradiation. BrUTP incorporation assay, a well-established assay complementary to EU labeling, allowed us to measure RNA transcription at 7, 15, and 30 min after irradiation. Depletion of CtIP did not significantly alter RNA transcription in non-damaged U2OS cells when assessed by BrUTP incorporation into non-nucleolar nascent RNA (Fig. 6a, b). As expected, global transcription decreased upon DNA damage in a CtIP-independent manner. Interestingly, our time-course experiments suggested that CtIP was required for the timely recovery of global cellular transcription after 30 min post-irradiation, while being dispensable for the generation of nascent RNA associated with DSB repair very early after irradiation (Fig. 6a, b). However, BRCA1-deficient cells showed reduced RNA transcription at all three post-irradiation time points examined, as well as under non-irradiated conditions (Supp. Fig. 9a, b), suggesting a possible replication- and/or transcription-associated function of BRCA1 at least partly independent of exogenous DSBs. In this context, we also assessed CtIP interaction with RNAPII upon DNA damage. Interestingly, CtIP interacted with RNAPII in non-irradiated as well as irradiated cells, yet such interaction was reduced during the initial phases post-IR, while recovering to pre-irradiation levels at 60 and 120 min after irradiation (Fig. 6c). Taken together with the other results of this study, we suggest that CtIP plays a dual role, first in the initiation phase and then later in progression of transcription-coupled DNA resection in DSB repair by the HR pathway.

## Discussion

One of the major recent advances in biomedicine has been the realization that diverse forms of RNA are intimately involved in a much broader range of fundamental biological processes than traditionally thought. RNAs and RNA:DNA hybrid structures have been widely implicated in physiological mechanisms and molecular pathogenesis of grave diseases such as cancer. Among the emerging roles of RNA is the causal involvement in various aspects of genome integrity maintenance, including repair of DNA DSBs, arguably the most hazardous genotoxic lesions^8,23,39^. Despite recent advances and studies on transcription-coupled DNA repair^11,12,40–42^, the outstanding issues of whether and mechanistically how are DSB-associated RNAs involved in the choice between NHEJ and the error-free HR pathways have remained unsolved.

**Fig. 7:**
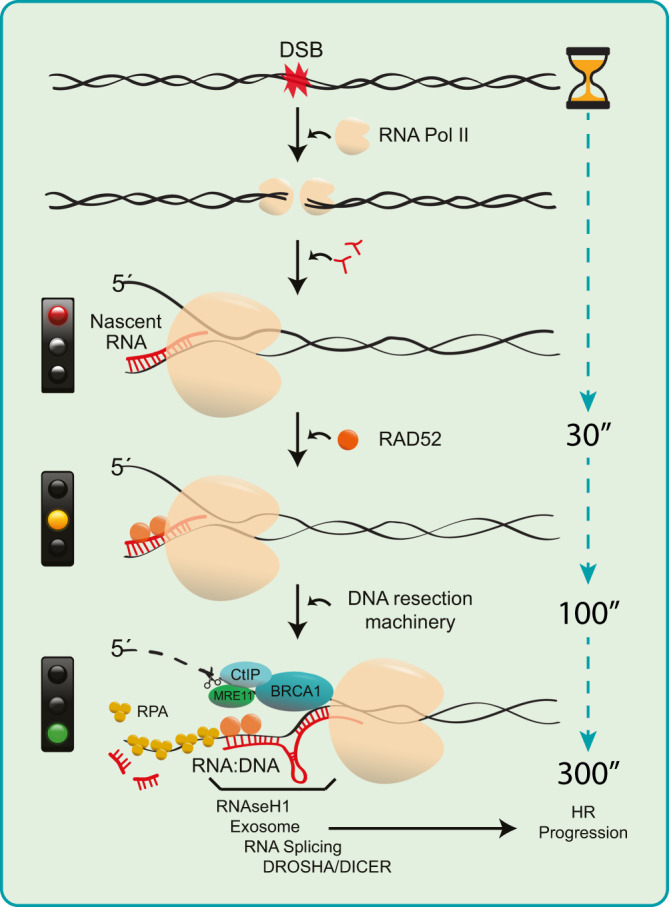
Schematic model of the regulation and role of RNAPII-generated nascent RNA to guide DNA end resection and DSB repair by HR. Transcriptionally active RNAPII is more prominent in S-G2 cell cycle phases (showed in Fig.1) when HR is known to repair DSBs. RNAPII recruitment to DSBs, that involves the pre-initiation complex (PIC)^23^, favors nascent RNA transcription, leading to generation of small RNA:DNA hybrid structures that cause RNAPII pausing (showed in Figs.2, 3). RAD52 and XPG are capable of rescuing the transiently paused RNAPII activity (8), allowing for 5’DNA strand displacement. Experimental inhibition of transcription impairs recruitment of the DNA resection factors CtIP, MRE11 and BRCA1 (showed in Figs.4, 5) to DSBs. MRE11 initiates 5’strand degradation, while the CtIP-BRCA1 axis is essential to regulate the speed of resection^23^ by controlling transcription upon DNA damage (showed in Fig.6). We show that CtIP and BRCA1 are factors promoting and/or re-starting the locally paused RNAPII-mediated transcription. These upstream events guide the DSB repair choice towards HR through initiation and progression of DNA end resection, in a feedback loop in which proper CtIP and BRCA1 recruitment are stimulated by the RNAPII at the DSB site, at least in part through complex formation between CtIP and RNAPII. Additional proteins and auxiliary processes such as exosomes^57^ RNA splicing^50^ and Drosha/Dicer^20^ contribute to regulation of RNA:DNA hybrids along the initiated HR pathway to adjust this process in a cell context- and time-dependent manner to safeguard genomic integrity.

Our present study complements and advances these efforts by providing a conceptual framework for, and mechanistic insights into, the role of nascent RNA transcripts and RNA:DNA hybrids at DSBs, as critical factors promoting DNA end resection and hence the upstream steps for the decision of human cells to repair DSBs by HR (see Fig. 7 for our proposed model). Indeed, our data demonstrate that RNAPII plays an essential role in this process. A vital prerequisite for addressing this issue was the development of our two single-molecule nucleic acid analysis methods that we present here: R-SMART and RL-SMART. These methods allowed us to assess the RNA-related DSB-repair events upon irradiation of human cells with a clinically relevant dose of 5 Gy, in an unbiased, genome-wide manner. Ionizing radiation generates global DNA damage in both transcriptionally active and silent genomic regions. Based on the current knowledge, we suggest that the bulk of such random IR-induced DSBs occur in otherwise non-transcribed chromatin because over 90% of the human genome is free from either protein-coding genes^16^ or non-protein-coding yet transcribed elements^43^. It is known that the global RNA transcription activity becomes inhibited upon DNA damage, and factors including ATM and cohesin contribute to this mechanism^17,44^. In our present study, RNA transcription silencing occurred within the initial 30 min upon irradiation, consistent with the published reports^9,17,45^. However, we show that nascent RNA formation is recovered by 60 min, and it is already increased by 30 min post-irradiation, compared to non-irradiated cells (Fig. 1 and Fig. 6), probably as a consequence of nascent RNA generated at DSB sites combined with the regular activity of RNAPII in the transcriptionally active genomic regions, the latter largely responsible for the nascent RNA detected also in the mock-treated cells. Within the initial 30 min post-irradiation the choice of DSB repair pathway is made, a process in which our results show the active regulation of RNAPII-mediated transcription at DSB sites is essential. Notably, our results also challenge the notion that HR-mediated repair might be limited to only those DSBs present in genomic loci that are transcriptionally active under physiological, non-stressed conditions^8^. The results from the R-SMART technique allowed us to conclude that nascent RNAs actively generated during DSB repair are linked to ssDNA resection tracts, implicating new RNA synthesis during HR (Fig. 2).

Furthermore, using the related RL-SMART approach, we observed that de novo transcription around DSB sites promotes the formation of RNA:DNA hybrid structures sensitive to resolution by RNAseH1 (Fig. 3) and possibly by other proteins such as Senataxin^19^ or XPG^8^. While the accumulation of some RNA:DNA hybrids can undermine genomic stability, RNA:DNA hybrids are also required for efficient DSB repair^4,7^. RNA:DNA hybrids commonly form when the RNAPII pauses during transcription^4^, thereby, in the context studied here, allowing recruitment of DDR factors to the DNA break. At the same time, RNA:DNA hybrid generation during the DSB-flanking RNA synthesis is primarily affected by depletion of CtIP or BRCA1 during the first 30 min after DNA damage. (Fig. 3). These results suggest that RNAPII recruitment to DSBs and acute bidirectional transcription^9^ results in the first round of RNA transcripts forming RNA:DNA hybrids and DNA resection factors becoming recruited after RAD52-dependent resolution of these hybrid structures (Fig. 7). Paused RNAPII activity needs to be re-launched after incorporation of DNA resection machinery into the DNA repair complex, and we show that efficient recruitment to DSBs of CtIP, a factor involved in DNA resection, requires active RNAPII (Fig. 4) as well as MRE11 for the early step of transcription-associated DSB repair. Furthermore, recruitment of HR factors such as RPA, BRCA1 and RAD52 to DSBs was also impaired after chemical inhibition of RNAPII, at the same time enhancing the recruitment of NHEJ-related proteins 53BP1 and RIF1. Indeed, PBAF-mediated transcription silencing flanking DNA breaks contributes to NHEJ^46^, and H4 deacetylation, which is associated with repression of transcription, favors 53BP1 foci formation early upon DNA damage^47^.

We also show that RNA synthesis inhibition impairs both the formation of RNA:DNA hybrids in the proximity of DSBs and HR repair, consistently with recent studies in yeast^7^ and mammalian models that however focused only on transcriptionally active regions of the genome^8,19,20^. Interestingly, while CtIP is essential to initiate DNA resection, CtIP’s precise mechanistic role in DNA resection has remained unclear. Our data indicate that CtIP, BRCA1 and their interplay are essential for re-launching the transiently paused activity of RNAPII and further progression of DNA resection (Fig. 7). Indeed, we suggest that DNA resection machinery recruitment and transcription initiation by RNAPII are mutually interdependent mechanisms, in that RNAPII is required to recruit HR factors to DSBs, and in turn the DNA resection factors promote transcriptional re-start at DSBs, in agreement with Domingo-Prim et al.^48^

Taken together with the current knowledge, our data, therefore, suggest the model (Fig. 7) in which RNAPII is actively recruited to the DSBs^21,23^, nascent RNA transcription by RNAPII induces the local formation of RNA:DNA hybrids that trigger RAD52 incorporation to resolve these structures^8^, together with RNAseH1 recruitment upon RPA coating of ssDNA^45^. RNAPII pausing occurs as a regulatory step in transcription, allowing time and local environment to resolve the DNA-RNA and provide a displaced 5′−3′ ssDNA available to recruit the resection machinery. CtIP, and its interaction with BRCA1^26,38^, promotes RNA transcription, permitting 5′−3′ degradation by exonucleases such as MRE11^49^. Regulation of RNA transcription through the CtIP-BRCA1 axis, removal of RNA:DNA hybrids, secondary RNA structures, RNA degradation and lncRNA for splicing machinery^50^, exosome^48^, and Dicer-Drosha^21^ complexes may all contribute to managing the later steps of HR, allowing the accrual of the RAD51-BRCA2^51^ cascade whose presence and the ensuing resolution of these various structures could be essential to avoid genome instability. Complementary to our present concept, work implicating nascent transcription in DSB repair yet focusing on the role of RNAPIII rather than RNAPII, has been published after the completion of our study^24^, thus independently validating the model we propose here.

In conclusion, nascent DSB-flanking RNAs and their local dynamics are essential for proper guidance of the cell’s decision to repair the potentially lethal DSB lesions via the faithful HR pathway, thereby safeguarding homeostasis and minimizing the development of pathologies, including cancer. Moreover, as cancer cells feature an enhanced load of endogenous DSBs^52–54^, our present concept has also implications for the emerging cancer treatments by transcription inhibitors^55,56^. This strategy likely exploits an emerging vulnerability of tumor cells whose survival and proliferation are ‘addicted’ to altered checkpoint signaling and dependent on repair mechanisms dealing with the excessive burden of chromosomal breaks.

## Methods

### Cell culture

U2OS and HeLa cells were cultured in Dulbecco’s modified eagle medium (DMEM) with high glucose plus GlutaMax supplemented with 10% FBS, 100 μg/ml streptomycin and 100 U/ml penicillin at 37 °C in 5% CO2. siRNAs against CtIP (GCUAAAACAGGAACGAAUC), BRCA1 (GGAACCUGUCTCCACAAAG), RAD52 (ThermoFisher, s11747), 53BP1 (GAAGGACGGAGTACTAATA) and a non-target control sequence (Sigma Aldrich) were transfected with the RNAiMax lipofectamine reagent mix (Life Technologies), according to the manufacturer’s instructions. Cells were cultured in the presence of THZ1 (Calbiochem, 5323720001), Triptolide (Sigma-Aldrich, T3652) or Pladionalide-B (CAS Number: 445493-23-2) using concentrations mentioned in each figure.

### Immunofluorescence

U2OS cells were grown on coverslips and pre-extracted for 3 min on ice using 0.2% Triton X-100 in PBS, then fixed with 4% paraformaldehyde (w/v) in PBS for 10 min, washed three times with PBS, and blocked for at least 1 h with 5% FBS diluted in PBS. Cells were incubated with adequate primary antibodies (Supplementary Material 1), diluted in 5% FBS in PBS for 16 h at 4 °C, washed with PBS, and then incubated with secondary antibodies (Supplementary Material 1) diluted in 5% FBS in PBS for 1 h at room temperature (RT). The cells were then washed twice with PBS and the coverslips were mounted with Vectashield mounting medium (Vector Laboratories) containing 4′,6-diamidino-2-phenylindole and analyzed using a LEICA microscope. At least 2000 cells per sample were scored. Experiments were repeated at least three times independently.

### High-content image acquisition

Quantitative image-based cytometry (QIBC) was performed as previously described^36^. The images were acquired in an automatic and unbiased way by scanR acquisition software 3.0 and analyzed by scanR image analysis software 3.0. The results were exported as txt files. The txt data set was further processed with spotfire and PRISM 8 software (Graphpad Software Inc) for further analysis. Statistical significance was determined with Ordinary one-way and two-way ANOVA tests using multiple comparison using PRISM 8 software (Graphpad Software Inc). Statistically significant differences were labeled with one, two, or three asterisks if *p* < 0.05, *p* < 0.01 or *p* < 0.001, respectively.

### Metabolic labeling of nascent RNA by EU

Nascent RNA was visualized using metabolic labeling using Invitrogen™ Click-iT™ RNA Alexa Fluor™ 594 imaging kit, with modifications. Briefly, cells were cultured in complete media and pulsed for 30 min with EU at a final concentration of 1 mM, before fixation with 4% paraformaldehyde (PFA) for 10 min. After fixation, cells were permeabilized with 0.5% Triton X-100 and washed 3 times with Tris buffered saline (TBS) (50 mM Tris pH 8.0, 150 mM NaCl). The click reaction master mix was then prepared as follows: 5 µM Alexa Fluor 488 azide, 2 mM CuSO4, 100 mM sodium ascorbate before sample preparation for acquisition using QIBC. The mean intensity of the nucleoli EU was analyzed using a spot detector tool and was excluded from the EU intensity of the nucleus to determine the EU intensity of the nucleoplasm.

### R-SMART

HeLa and HeLa-RNAseH1 cells down-regulated for the indicated genes were grown in the presence of 10 μM bromodeoxyuridine (BrdU, GE Healthcare) for 24 h. The cultures were then irradiated (5 Gy) and incubated with 100 μM RNA precursor 5-ethynyluridine (EU) and harvested after 30 min. Cells were lysed using Spreading Buffer (200 mM Tris:HCl pH 7.5, 50 mM EDTA, 0,5% SDS). 2000 cells were used to stretch nucleic acid fibers on coverslips using a 15° angle of incline for 8 min and fixed with cold methanol:acetic acid (3:1) for 10 min at room temperature. Five slides were stretched for all experimental conditions and two or three slides for each condition were stained. Nascent RNA was detected using the Click-it RNA Alexa Fluor 488 imaging kit (ThermoFisher Scientific) following the manufacturer’s instructions. The samples were then incubated directly without denaturation with an anti-BrdU mouse monoclonal antibody (Becton Dickinson, 347580). Secondary antibodies were DayLight 555 antimouse (Thermo Fisher Scientific).

DNA fiber images were acquired using an LSM800 confocal microscope (Carl Zeiss) and a Plan-Apochromat 63×/1.4 numerical aperture (NA) oil immersion objective (Carl Zeiss). The labeled RNA and DNA fibers were analyzed using LSM800 Zeiss ZEN software (Blue edition), using a colocalization tool to measure pixel staining for both fluorescence. More than 20 fields or more than 100 fibers were scored during measurements on each slide for each repeated experiment. The percentage of RNA and DNA staining pixels and their colocalization was presented from the experiments.

### RL-SMART

HeLa and HeLa-RNAseH1 cells were treated with 10 μM bromodeoxyuridine (BrdU, GE Healthcare) for 24 h. The cultures were then irradiated (5 Gy) harvested after 1 h. Cells were lysed using Spreading Buffer (200 mM Tris:HCl pH 7.5, 50 mM EDTA, 0,5% SDS). 2000 cells were used to stretch and fix nucleic acid fibers as described in the R-SMART section. The samples were then incubated directly without denaturation with an anti-BrdU rat monoclonal antibody (Becton Dickinson, 347580) and a S9.6 mouse monoclonal antibody (Kerafast, ENH001). Secondary antibodies were DayLight 550 anti-rat and 488 antimouse. Five slides were stretched for all experimental conditions and two or three slides for each condition were stained. Images were taken as described above for the R-SMART technique. The percentage of pixels of RNA:DNA hybrids and DNA resection staining, and their colocalization, were analyzed as in the R-SMART technique and presented from both experiments. High resolution images were acquired with STELLARIS 8 Confocal Microscope (Leica Microsystems).

### DNA Damage by laser microirradiation

Cells were seeded in 1 glass bottom well chamber one day before analysis and pre-sensitized with 10 μM bromodeoxyuridine (BrdU, GE Healthcare) for 24 h. Before laser irradiation, cells were incubated with 1 µM THZ1 (1604810-83-4) for 1 h, at 37 °C and 5% CO2. The cells were then maintained under the same conditions using the Temperature Control Chamber (PerkinElmer UltraView VoX), and images were taken with a Nikon Eclipse Ti microscope equipped with a 63x oil objective and equipped with a Perkin Elmer spinning disk. Images were collected every 4 s for 10 min after irradiation. Cellular nuclei were irradiated with a 355 nm UV ablation laser at a power setting of 0.15, a repetition rate of 200 Hz, a pulse energy >60 μJ, pulse length< 4 ns (Rapp OptoElectronic). The intensity of the GFP signal was measured using ImageJ software for at least 20 cells per condition from three biological replicates.

### In situ labeling of newly synthesized RNA with BrUTP incorporation assay

Cells were seeded on coverslips and on the day of the BrUTP incorporation assay were 70-75% confluent. Cells were first irradiated and incubated for the indicated period of time (7, 15, or 30, respectively). Coverslips were washed once with PBS at each of the above time points and incubated with permeabilization buffer (20 mM Tris-HCl, pH 7.4, 5 mM MgCl2, 0.5 mM EGTA, 25% glycerol, 0.05% Triton X-100, 1 mM PMSF and ribonuclease inhibitor 20 U/ml) for 2 min at RT for every time point. Next, the permeabilization buffer was removed from the coverslips and transcription buffer (20 mM Tris-HCl, pH 7.4, 5 mM MgCl2, 0.5 mM EGTA, 25% glycerol, 1 mM PMSF, 100 mM KCl, 20 U/ml ribonuclease inhibitor, 500 μM BrUTP, 500 μM CTP, 500 μM GTP and 2 mM ATP) was added. The coverslips were incubated for additional 8–10 min at 37 °C. After removing the transcription buffer, the coverslips were gently washed with cold PBS and fixed in 4% paraformaldehyde for 10 min at RT. Indirect immunofluorescence was performed using mouse anti-BrdU antibody (BD347580) to detect the incorporation of the BrUTP analog in nascent transcripts. The images were acquired using an LSM800 confocal microscope (Carl Zeiss) and a Plan-Apochromat ×63/1.4 numerical aperture (NA) oil immersion objective (Carl Zeiss). The labeled nascent RNAs were analyzed using LSM800 ZEN software (Blue edition). The mean intensity of BrUTP incorporation was measured via ZEN blue (Zeiss) at different sites of the nucleus of the cell avoiding the nucleolar compartment. Data were processed using PRISM 8 software (Graphpad software Inc.) and statistical significance was determined using multiple comparison with Ordinary One-Way ANOVA.

### Immunoblotting

Whole-cell line extracts were prepared in Laemmli buffer (4% SDS, 20% glycerol and 125 mM Tris-HCl, pH 6.8), and proteins were resolved using SDS-PAGE and transferred to nitrocellulose membranes, followed by immunoblotting. Western blot analysis was performed using the antibodies listed in Table 1. Results were acquired using the ChemiDoc system and visualized with Image Lab software 5.1 (Bio-Rad).

**Table 1:** List of antibodies

Antibodies used	Company	Catalog	Host	DILUTION
BrdU	Apbiotech	RPN20AB(RPN202)	Rat	1:500
BrdU	Bd biosciences	347580	Mouse	1:500
S9.6	Kerafast	ENH001	Mouse	1:250
53BP1	Millipore	MAB3802	Mouse	1:500
S139-H2AX	Millipore	16-193	Mouse	1:1000
S139-H2AX	Cell signaling	S2577s	Rabbit	1:500
BRCA1	Santa cruz	sc-6954	Mouse	1:200
RNAPII	Abcam	ab817	Mouse	1:2000
S5P-RNAPII	Abcam	ab5131	Rabbit	1:500
CtIP	Active motif	61141	Mouse	1:500
RAD52	Santa cruz	sc-365341	Mouse	1:500
α-TUBULIN	Gene tex	GTX628802	Mouse	1:2000
S4/S8 RPA	Bethyl	A300-245A	Rabbit	1:500
RAD51	Abcam	ab63801	Mouse	1:200
PCNA	Immuno concepts	2037	Human	1:1000
RIF1	Bethyl	A300-569A	Rabbit	1:500
ALEXA-FLUOR 488 GOAT	Invitrogen	A11029	Mouse	1:1000
ALEXA-FLUOR 568 GOAT	Invitrogen	A11036	Rabbit	1:1000
ALEXA-FLUOR 555 GOAT	Invitrogen	A21424	Mouse	1:1000
ALEXA-FLUOR 647 GOAT	Invitrogen	A21445	Human	1:1000
ALEXA-FLUOR 568 GOAT	Invitrogen	A11077	Rat	1:1000

### Co-immunoprecipitation assay

U2OS cells were irradiated and incubated for specific time points at 7, 15, 30, 60, and 120 min. Nuclear protein fractions were prepared using the Nuclear Complex co-IP kit (Active Motif #5400), according to the manufacturer. Subsequently, the nuclear extracts were diluted in the appropriate volume of IP buffer (0.5% NP40, 50 mM Tris-HCl, pH 7.4, 150 mM NaCl, 1 mM EDTA) supplemented with protease and phosphatase inhibitors. The diluted nuclear extracts were incubated with anti-RNA polymerase II CTD repeat YSPTSPS antibody (8WG16)- ChIP grade (Abcam, ab817) overnight at 4 °C. The protein extracts were then incubated with 25 μl of protein G dynabeads (Thermo Fisher, 10004D) for 1 h at 4 °C. The beads were washed 5 times with 250 μl of IP buffer and 20 μl of Laemmli sample buffer was added combined with heating at 97 °C for 5 min to elute the precipitated protein fraction from the beads. Western blot analysis was performed using the antibodies listed in Table 1. Semi-quantification of band density was carried out by image J.

### Reporting summary

Further information on research design is available in the Nature Research Reporting Summary linked to this article.

## Supplementary information

Supplementary Information

Description of Additional Supplementary Files

Supplementary Movie 1

Reporting Summary

## Data Availability

The data generated during this study are available from the corresponding author upon reasonable request. Source data are provided with this paper.

## Source data

Source Data
