# Supplementary material for: CtIP-dependent nascent RNA expression flanking DNA breaks guides the choice of DNA repair pathway
Source: Nat Commun. 2022 Sep 9;13:5303. doi: 10.1038/s41467-022-33027-z (PMC9463442; doi:10.1038/s41467-022-33027-z)
Supplement: Supplementary file 1 — Supplementary Information [file 41467_2022_33027_MOESM1_ESM.pdf]

CtIP-dependent nascent RNA expression flanking DNA breaks guides the choice of DNA repair pathway

Daniel Gómez-Cabello*, George Pappas, Diana Aguilar-Morante, Christoffel Dinant and Jiri Bartek*.

***Correspondence:** dgcabello@us.es and jb@cancer.dk

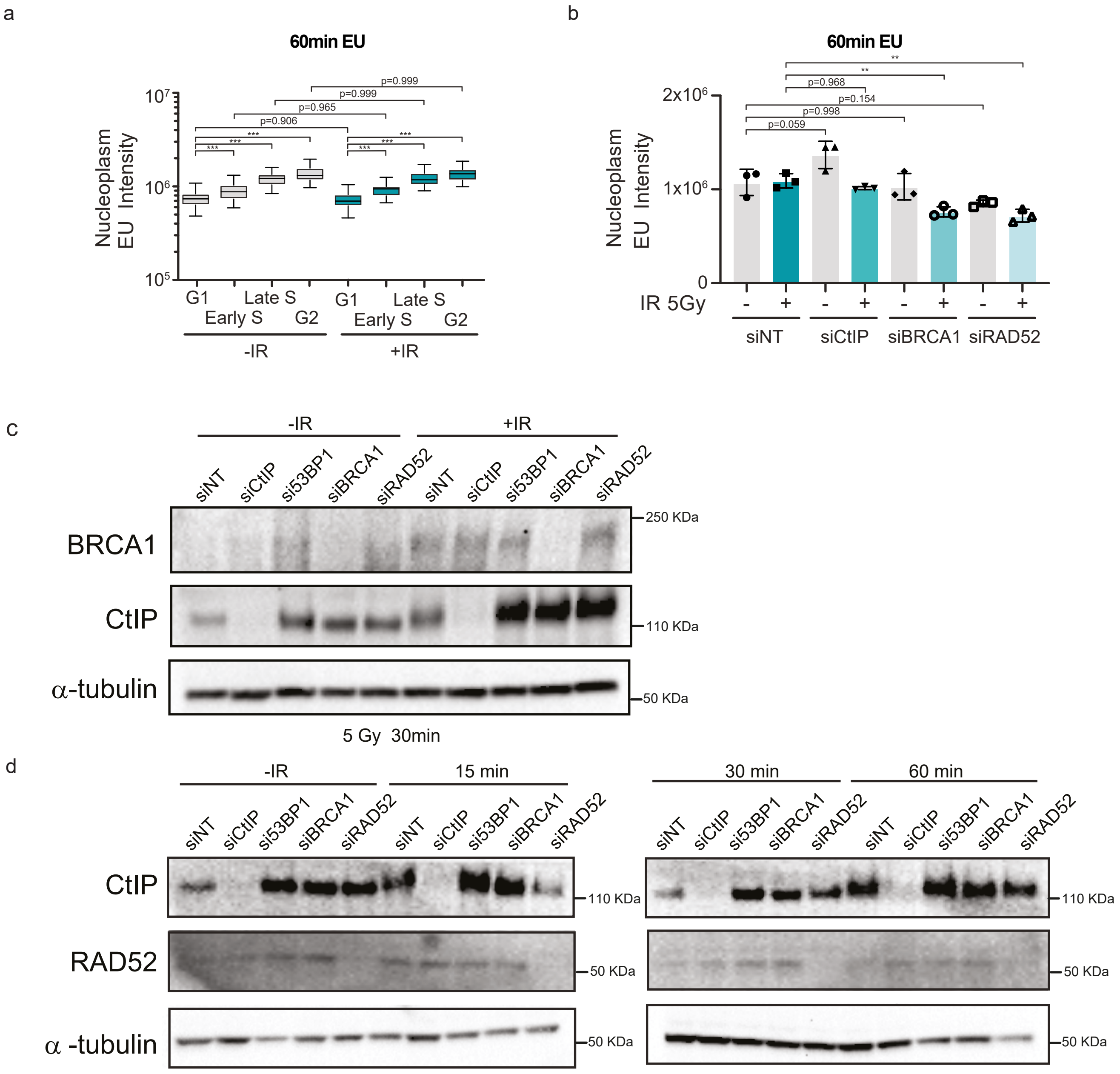
a
60min EU
Nucleoplasm EU Intensity
10^7
10^6
10^5
p=0.999
p=0.999
p=0.965
p=0.906
***
G1
Early S
Late S
G2
-IR
+IR
b
60min EU
Nucleoplasm EU Intensity
2x10^6
1x10^6
0
**
p=0.968
p=0.154
p=0.998
p=0.059
IR 5Gy
siNT
siCtIP
siBRCA1
siRAD52
c
-IR
+IR
siNT
siCtIP
si53BP1
siBRCA1
siRAD52
BRCA1
CtIP
α-tubulin
250 KDa
110 KDa
50 KDa
5 Gy 30min
d
-IR
15 min
30 min
60 min
CtIP
RAD52
α -tubulin
110 KDa
50 KDa

Supplementary Fig. 1. Nascent RNA connection with DNA resection machinery.

a Graph shows nucleoplasm EU intensity in different cell cycle phases in non- and irradiated-U2OS cells. EU component was added upon DNA damage using 5Gy in irradiated cells and labelled for 60 minutes in both cellular conditions. Bar plots show the median (center), 25-75 percentile (box), and 5-95 percentile (whisker) from at least n=500 cells of 3 independent experiments. P values were calculated using multiple comparison with ordinary One-Way ANOVA. *** p>0.001.

b Histogram represents nucleoplasm EU intensity using siRNAs against indicated genes in non- and irradiated-cells. EU component was added upon DNA damage using 5Gy in irradiated cells and labelled for 60 minutes in both cellular conditions. Showing mean values and ± s.e.m from 3 independent experiments. P values were calculated using multiple comparison with ordinary Two-Ways ANOVA. **p= 0.0099 (siNT +IR vs siBRCA1 +IR), **p=0.0036 (siNT +IR vs siRAD52 +IR).

c immunoblot shows level of BRCA1, CtIP and α-tubulin proteins of irradiated (5 Gy) and non-irradiated U2OS cells after 48 hours of depletion of the correspond genes. Representative images are shown of 3 independent experiments.

d U2OS cells in presence of siRNAs against the showed genes upon irradiation (5 Gy) at different time points were blotted for CtIP, RAD52 and α-tubulin. Representative images are shown of 3 independent experiments.

Source data are provided as a Source data file.

Nascent RNA and DNA resection intensity upon 5 Gy irradiation

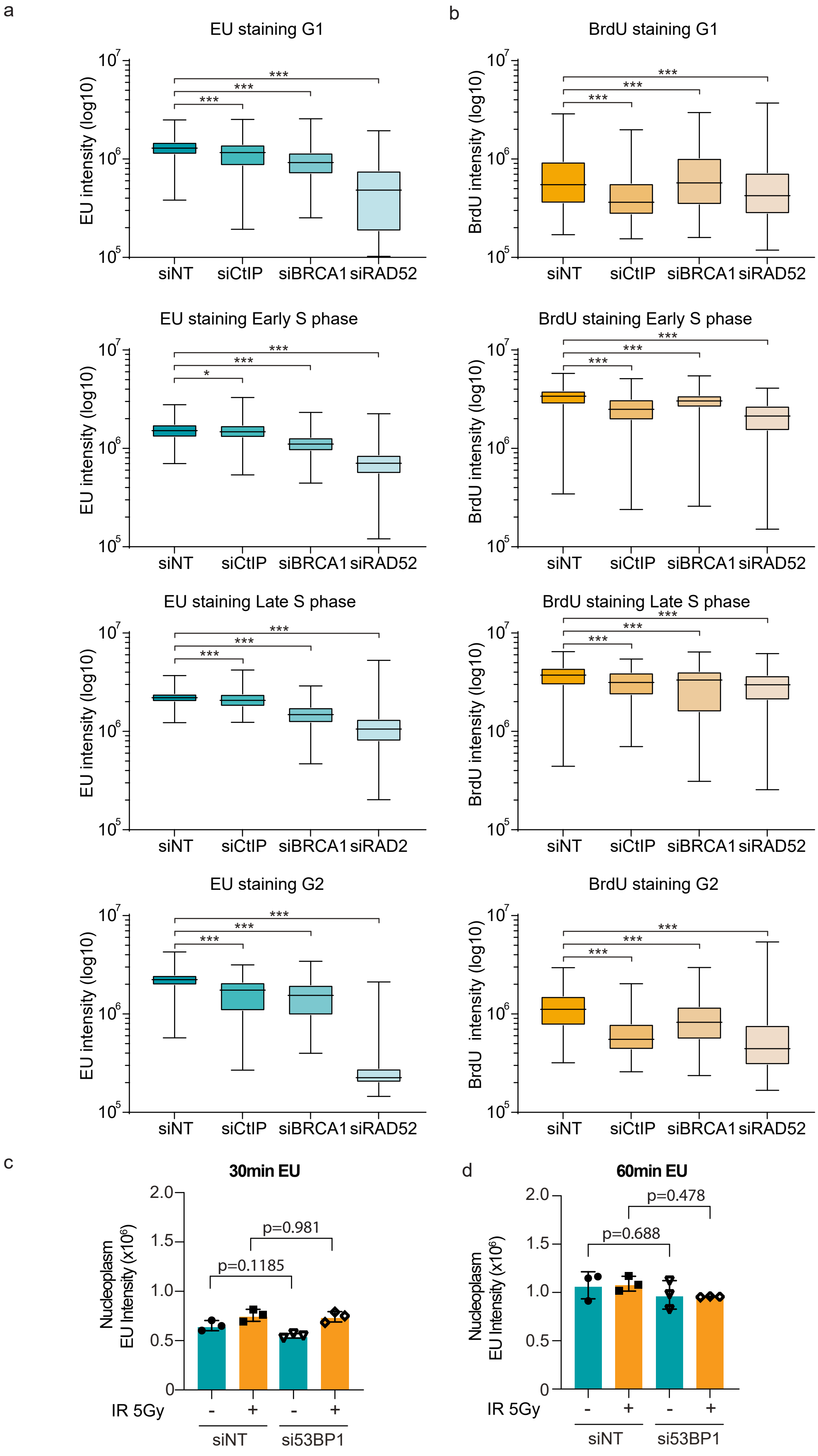

Supplementary Fig. 2. Nascent RNA transcription under DNA resection defects in the cell cycle phases.

a Graphs show nucleoplasm EU intensity in different cell cycle phases in irradiated-U2OS cells. EU component was added upon DNA damage using 5Gy and labelled for 30 minutes.

b Graphs show BrdU intensity in cell cycle phases in U2OS cells treated with BrdU during 24h at the same condition that (a) and BrdU labeling to measure intensity.

a-b Bar plots show the median (center), 25-75 percentile (box), and 5-95 percentile (whisker) from at least n= 800 cells of 3 independent experiments. P values were calculated using multiple comparison with ordinary One-Way ANOVA. *p=0.0322 and *** p>0.001

c-d Histogram shows nucleoplasm EU intensity in non- and irradiated-U2OS cells with siNT and siRNA against 53BP1. EU component was added upon DNA damage using 5 Gy in irradiated cells and labelled for 30 (c) and 60 minutes (d) in both cellular conditions. Showing mean values from 3 independent experiments. Error bars represent s.e.m. P value were calculated using multiple comparison with ordinary One-Way ANOVA.

Source data are provided as a Source data file.

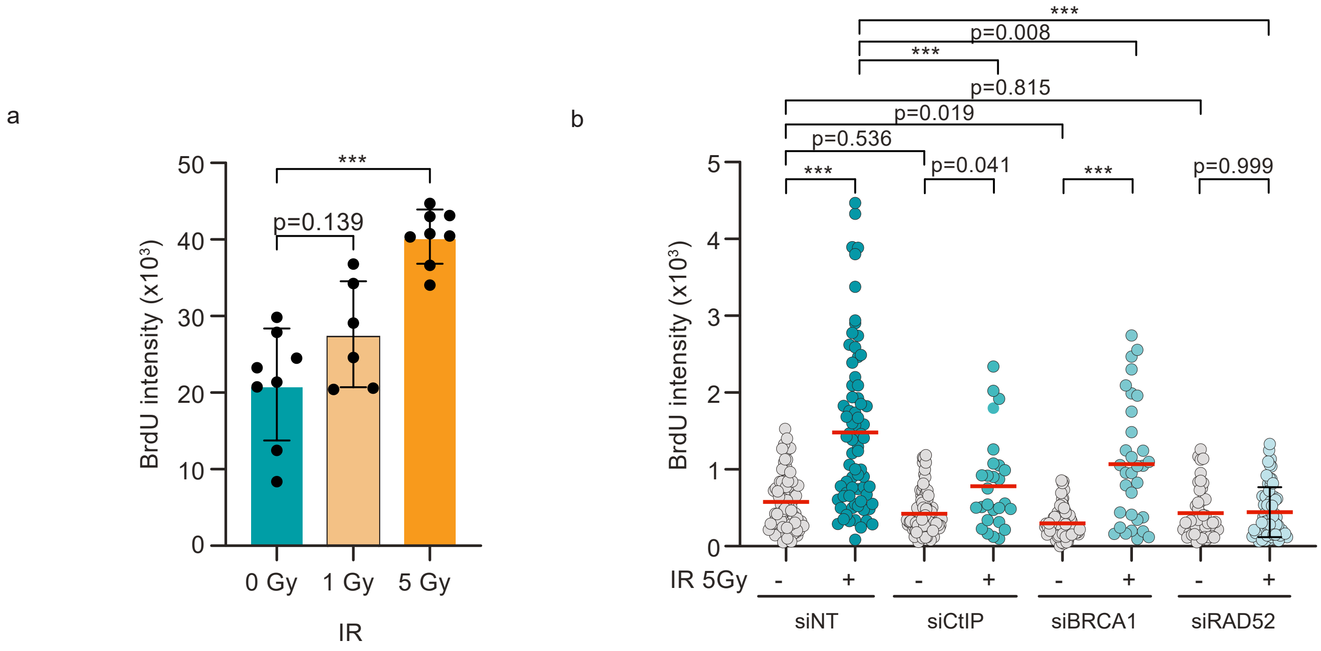
a
BrdU intensity (x10³)
50
40
30
20
10
0
***
p=0.139
0 Gy
1 Gy
5 Gy
IR
b
BrdU intensity (x10³)
5
4
3
2
1
0
***
p=0.008
***
p=0.815
p=0.019
p=0.536
p=0.041
***
p=0.999
IR 5Gy
-
+
siNT
siCtIP
siBRCA1
siRAD52

Supplementary Fig. 3. DNA resection detection using the R-SMART technique.

a Graph shows average of BrdU intensity from R-SMART samples. Measuring was taken from 0, 1 and 5 Gy samples previously incubated with BrdU 10 μM during 24 hours in HeLa cells. Showing mean values from 3 independent experiments. Data are presented as mean values ± s.e.m. P values were calculated using multiple comparison with ordinary One-Way ANOVA. ***p<0.0001.

b Plot showing BrdU intensity of samples from Hela cells treated with siRNA against CtIP, BRCA1 and RAD52. Measuring was taken from 0 and 5 Gy samples previously incubated with BrdU during 24 hours. At least 25 fields from 3 independent experiments were quantified. Data are presented as mean values ± s.e.m. P values were calculated using multiple comparison ordinary One-Way ANOVA. *** p>0.001

Source data are provided as a Source data file.

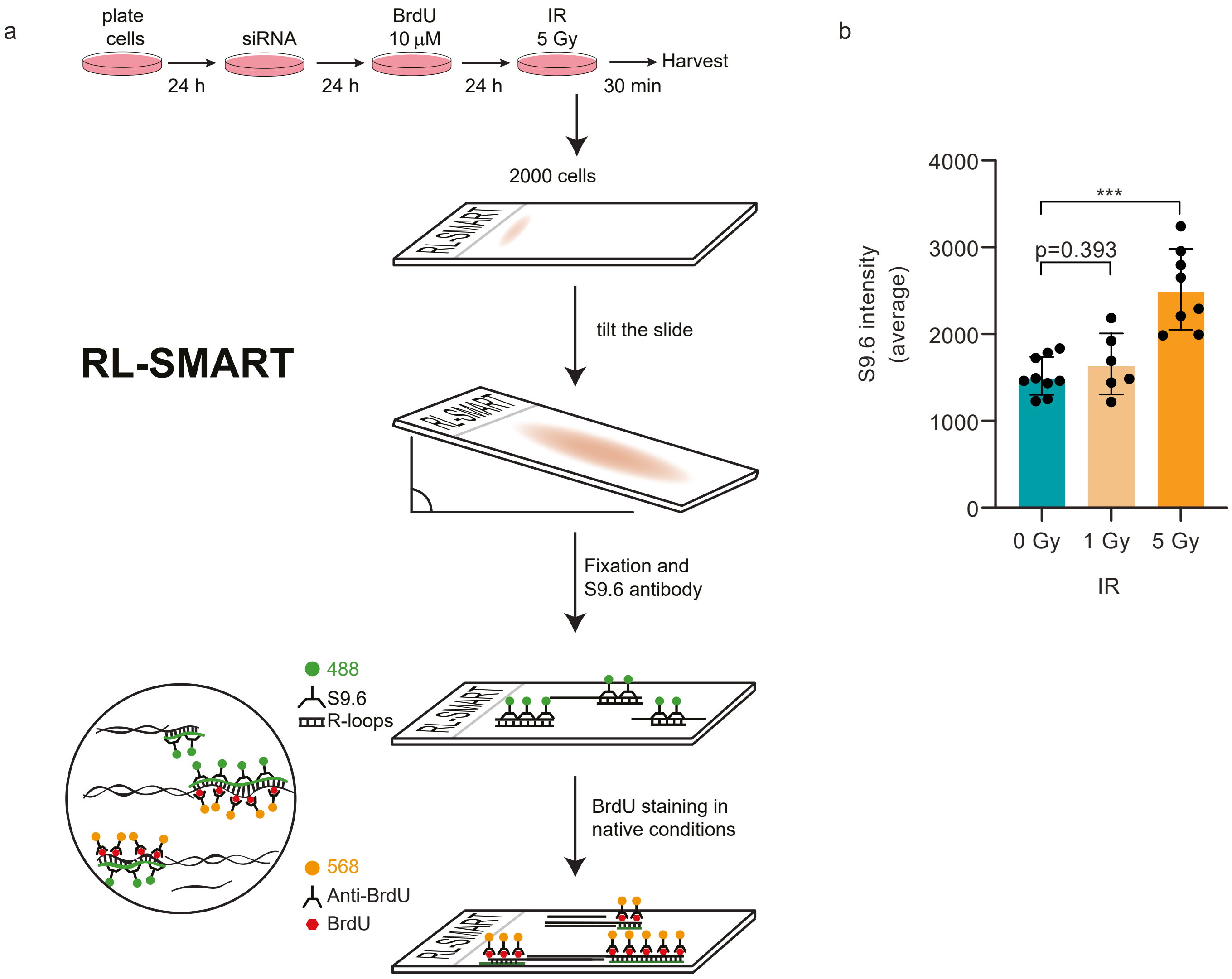
a
plate cells
siRNA
BrdU 10 µM
IR 5 Gy
Harvest
24 h
24 h
24 h
30 min
2000 cells
RL-SMART
tilt the slide
RL-SMART
Fixation and S9.6 antibody
488
S9.6
R-loops
BrdU staining in native conditions
568
Anti-BrdU
BrdU
b
S9.6 intensity (average)
4000
3000
2000
1000
0
***
p=0.393
0 Gy
1 Gy
5 Gy
IR

Supplementary Fig. 4. RL-SMART representation to measure RNA:DNA hybrids.

a Representative scheme of RL-SMART technology.

b Graph shows average of S9.6 intensity from RL-SMART samples irradiated with 0, 1 and 5 Gy. Showing mean values from more than 5 samples of 3 independent experiments. Data are presented as mean values ± s.e.m. and p values were calculated using multiple comparison with ordinary One-Way ANOVA. ***$p<0.0001$.

Source data are provided as a Source data file.

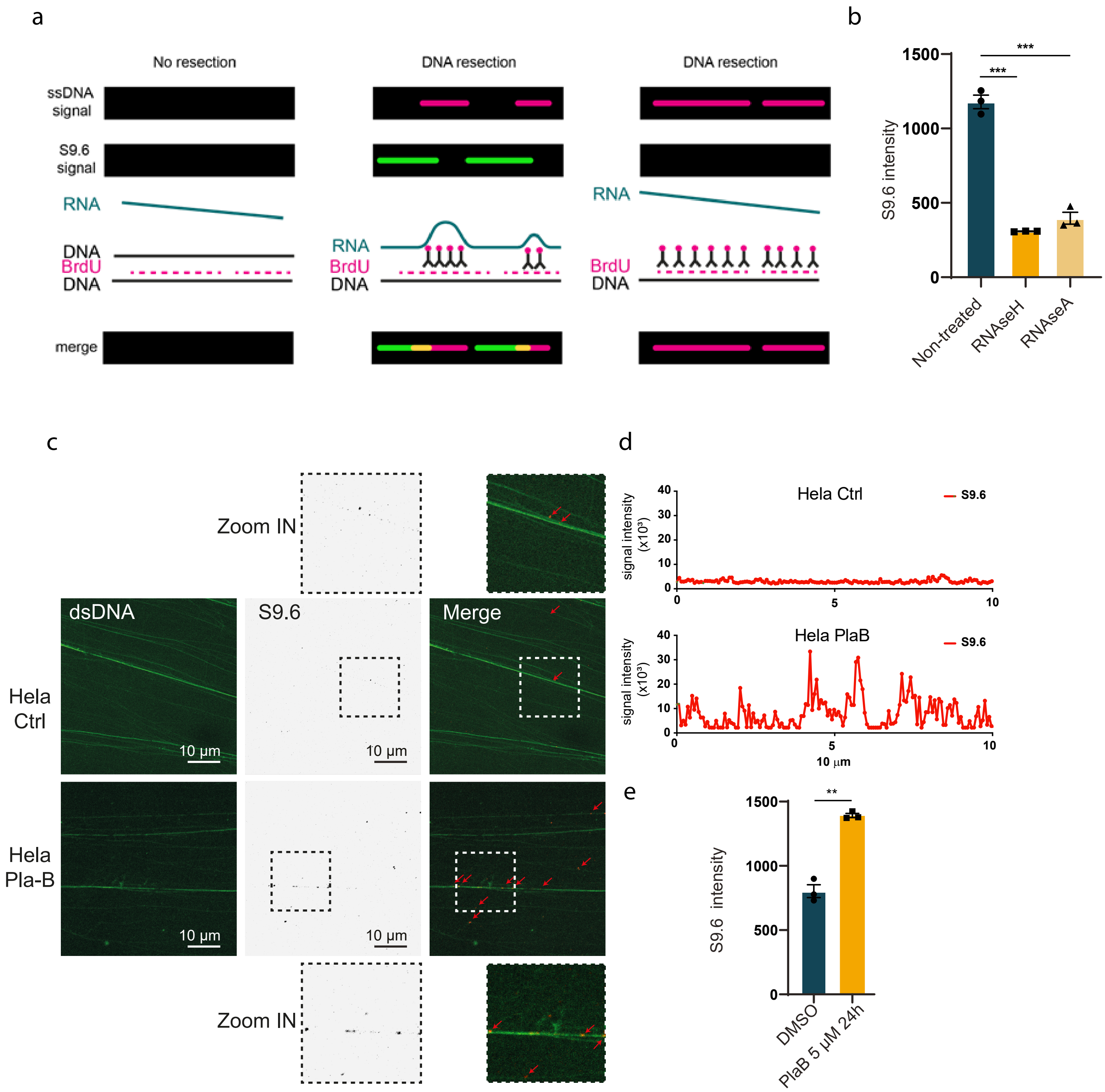
a
No resection
DNA resection
DNA resection
ssDNA signal
S9.6 signal
RNA
DNA
BrdU
DNA
merge
b
S9.6 intensity
1500
1000
500
0
***
***
Non-treated
RNAseH
RNAseA
c
Zoom IN
dsDNA
S9.6
Merge
Hela Ctrl
Hela Pla-B
10 µm
d
Hela Ctrl
Hela PlaB
S9.6
signal intensity (x10³)
40
30
20
10
0
5
10
10 µm
e
S9.6 intensity
1500
1000
500
0
**
DMSO
PlaB 5 µM 24h

Supplementary Fig. 5. RL-SMART technique is sensitive to Pladienolide-B for RNA:DNA hybrids detection. R-SMART and RL-SMART techniques to detect structures in DNA fibers after treatments.

a. Schematic representation showing different structures visualized using R-SMART and RL-SMART techniques.

b. Bar graph, RNA:DNA hybrid signals are removed upon RNAseH and A treatment upon nucleic acid spreading, respectively. Data are presented as mean values ± s.e.m of 3 independent experiments. P values were calculated using multiple comparison ordinary One-Way ANOVA. *** $p>0.001$.

c. Confocal microscope images of non- and Pladienolide-B-treated HeLa cells for RNA:DNA hybrid detection using the RL-SMART technique. Images are amplified for S9.6 detection and merged with dsDNA staining. Representative images are shown of 3 independent experiments. Scale bar: 10μm.

d. Representative profiles of S9.6 detection in a dsDNA (Yo-Yo-1 iodide labeling) observing a signal increase of RNA:DNA hybrid intensity in Pladienolide-B treated cells.

e. Quantification of S9.6 staining in nucleic acid fibers under conditions mentioned previously in d. Data are presented as mean values ± s.e.m using Two-tailed t test. **$p=0.004$

Source data are provided as a Source data file.

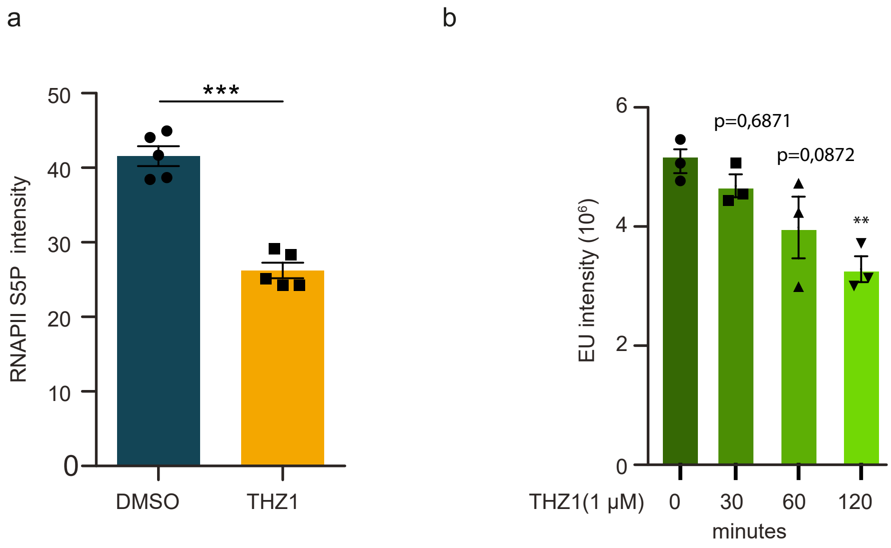
a
RNAPII S5P intensity
50
40
30
20
10
0
***
DMSO
THZ1
b
EU intensity (10^6)
6
4
2
0
p=0,6871
p=0,0872
**
THZ1(1 μM)
0
30
60
120
minutes

Supplementary Fig. 6. THZ1 inhibits Serine-5 phosphorylation of the RNAPII and synthesis of nascent RNA.

a Graph showing intensity of S5P-RNAPII after 2 hours of THZ1 1μM treatment as RNAPII inhibition test. Quantification shows 5 independent experiments. Mean values and ± s.e.m. are represented. P values were calculated using Two-tailed t test. *** $p>0.001$

b Graph shows EU intensity after THZ1 treatment for 0, 30, 60 and 120 minutes. Quantification shows the mean values from 3 independent experiments. Error bars represent ± s.e.m and p value were calculated using multiple comparison ordinary One-Way ANOVA. *$p=0.0089$

Source data are provided as a Source data file.

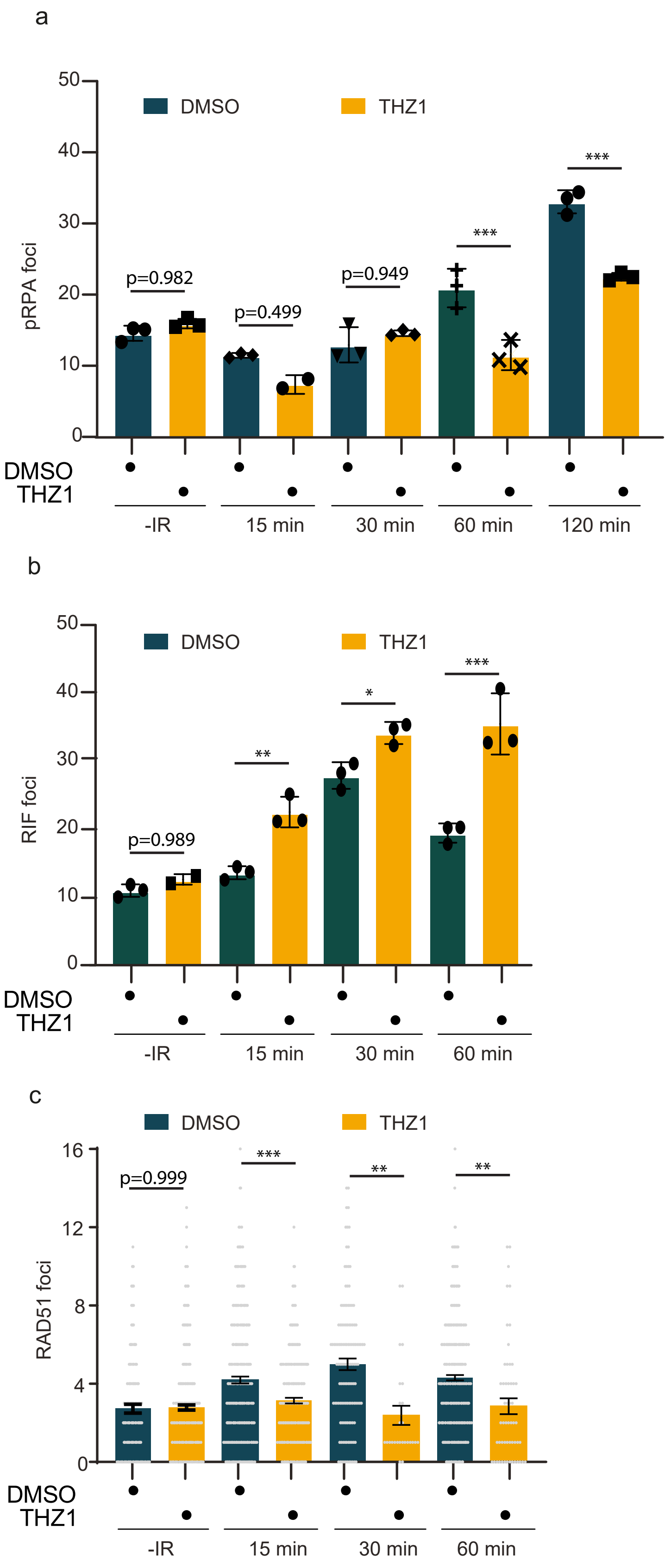

a
pRPA foci
DMSO
THZ1
p=0.982
p=0.499
p=0.949
***
***
DMSO
THZ1
-IR
15 min
30 min
60 min
120 min
b
RIF foci
DMSO
THZ1
p=0.989
**
*
***
DMSO
THZ1
-IR
15 min
30 min
60 min
c
RAD51 foci
DMSO
THZ1
p=0.999
***
**
**
DMSO
THZ1
-IR
15 min
30 min
60 min

Supplementary Fig. 7. THZ1 treatment impairs foci formation of homologous recombination factors.

a Graph showing S4/S8 phosphorylation of RPA foci upon 5 Gy-irradiation in U2OS cells previously treated with DMSO and THZ1 (1 μM for 2 hours). Bar graph for pRPA foci quantification at 0, 30, 60 and 120 minutes after irradiation. At least n=2000 cells examined over 3 independent experiments were quantified. Data are presented as mean values ± s.e.m and p values. ***p<0.0001.

b Graph showing RIF1 foci upon 5 Gy-irradiation in U2OS cells previously treated with DMSO, THZ1 (1 μM for 2 hours). Bar graph for RIF1 foci quantification at 0, 15, 30 and 60 minutes after irradiation. Data are presented as mean values ± s.e.m. and p values. *p=0.049 , **p=0.003 and ***p<0.0001.

c Scatter blot with bars showing RAD51 foci upon 5 Gy-irradiation in U2OS cells previously treated with DMSO, THZ1 (1 μM for 2 hours). Showing mean values and r RIF1 foci quantification at 0, 15, 30 and 60 minutes after irradiation. Data represented as mean values and ± s.e.m from at least n=200 cells of 3 independent experiments. **p=0.007, **p=0.01 and ***p<0.0001.

a-c At least n=2000 cells examined over 3 independent experiments were quantified. Data are presented as mean values ± s.e.m. P values were calculated using multiple comparison with ordinary One-Way ANOVA.

Source data are provided as a Source data file.

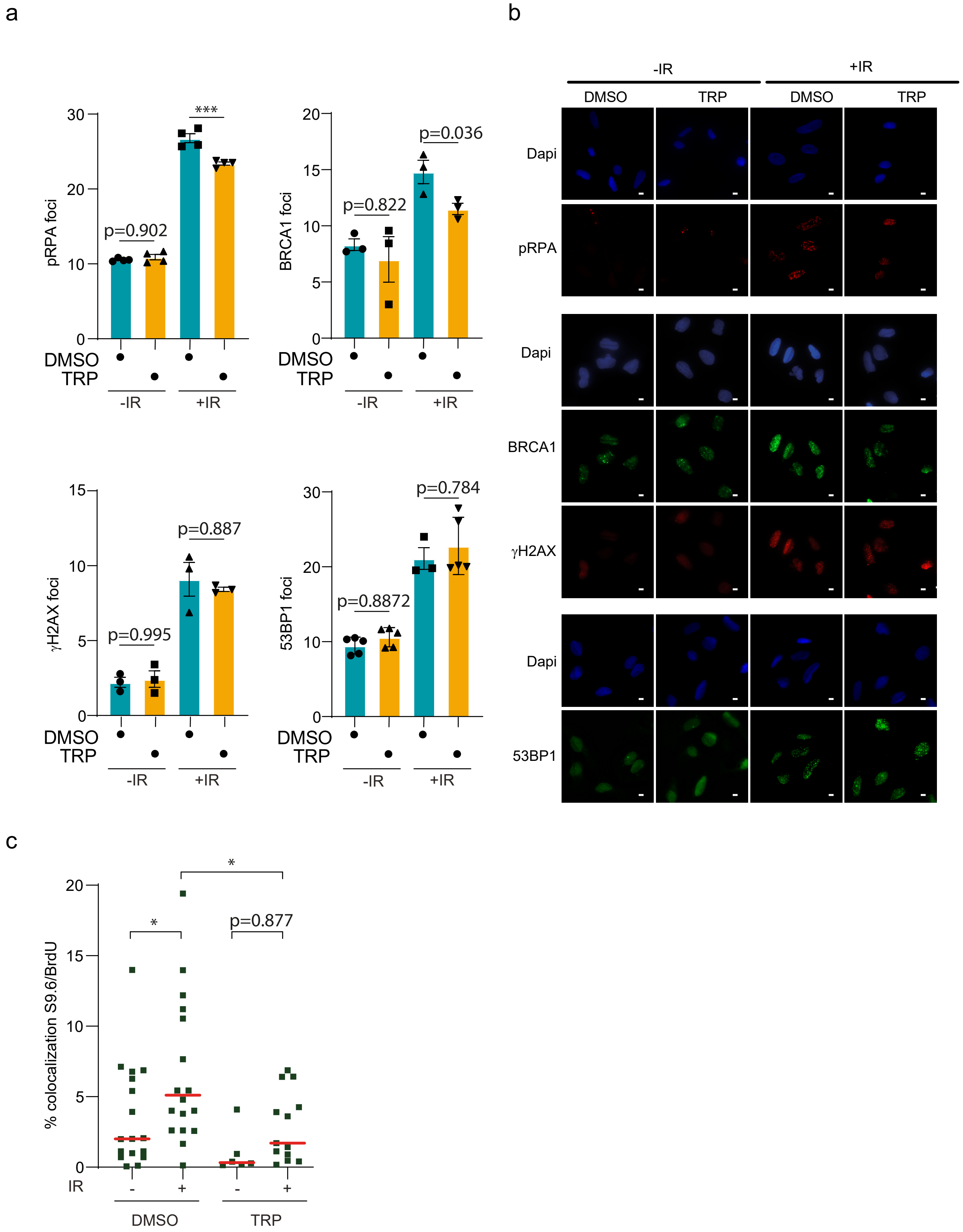
a
pRPA foci
p=0.902
***
BRCA1 foci
p=0.822
p=0.036
γH2AX foci
p=0.995
p=0.887
53BP1 foci
p=0.8872
p=0.784
DMSO
TRP
-IR
+IR
b
-IR
+IR
DMSO
TRP
Dapi
pRPA
BRCA1
γH2AX
53BP1
c
% colocalization S9.6/BrdU
*
p=0.877
IR
DMSO
TRP

Supplementary Fig. 8. Triptolide treatment impairs foci formation of homologous recombination factors.

a Graphs show S4/S8 phosphorylation of RPA, BRCA1, 53BP1 and S139 phosphorylation of H2AX foci fixed at 1 hour upon 5 Gy-irradiation in U2OS cells treated with DMSO and Triptolide (10 µM for 30 minutes). Data are presented as mean values ± s.e.m from 3 independent experiments and p value was calculated using multiple comparison with One-Way ANOVA. ***p=0.0002 (RPA foci), **p=0.036 (BRCA1 foci).

b Representative images of immunofluorescence for all DDR markers quantified in a. Scale bar: 10µm. N=3 independent experiments.

c. Dot graph shows percentages of RNA:DNA hybrids (S9.6) and ssDNA (BrdU) signal colocalization on resection tracts generated in non- and 5 Gy-irradiated Hela cells at 30 minutes after treatment with the indicated RNAPII inhibitors, THZ1 (1 µM -2 hours) and TRP (10 µM, 30 minutes), respectively. N=3 independent experiments. P value was calculated by multiple comparison with Ordinary One Way ANOVA. *p=0.041 and *p=0.040.

Source data are provided as a Source data file.

a

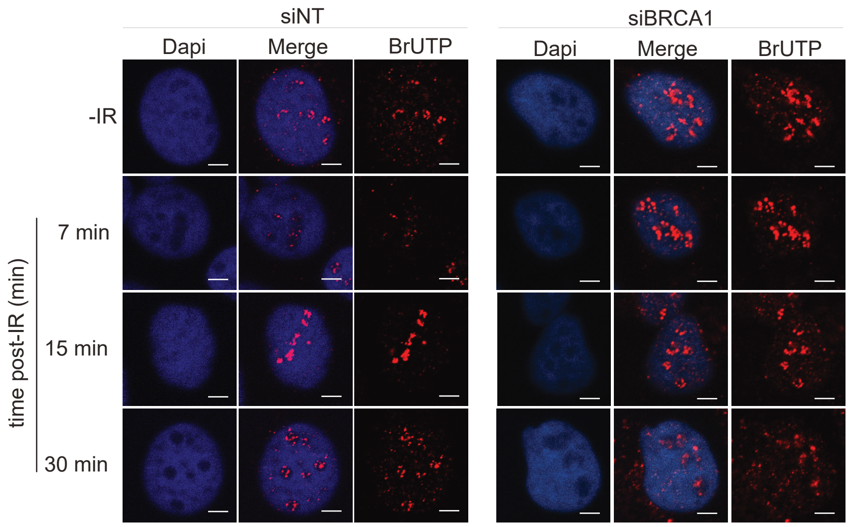
siNT
siBRCA1
Dapi
Merge
BrUTP
-IR
7 min
15 min
30 min
time post-IR (min)

b

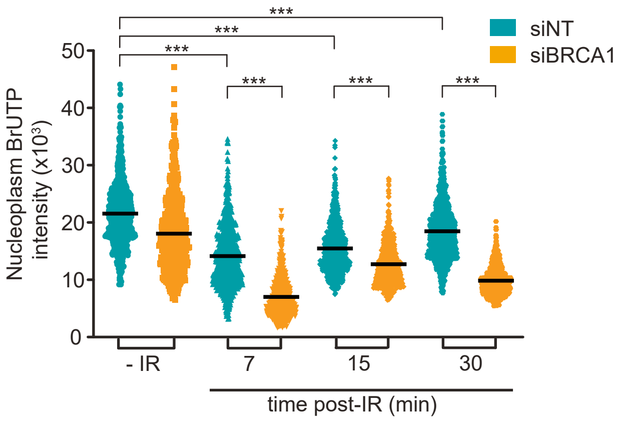
Nucleoplasm BrUTP intensity (x10³)
50
40
30
20
10
0
***
- IR
7
15
30
time post-IR (min)
siNT
siBRCA1

Supplementary Fig. 9. BRCA1 knockdown prevents transcription re-start after irradiation.

a Representative images of de novo transcription: BrUTP incorporation into nascent RNA at 7, 15 and 30 minutes post IR (5Gy) in BRCA1-depleted U2OS cells. Scale bar: 10µm. n=3 independent experiments.

b Dot plot shows nucleoplasm quantification of BrUTP incorporation under the experimental conditions cued in (a). At least n=200 cells examined over 3 independent experiments were quantified. P values were calculated using multiple comparison with ordinary One-Way ANOVA. *** p>0.001

Source data are provided as a Source data file.
