# Supplementary material for: CtIP-dependent nascent RNA expression flanking DNA breaks guides the choice of DNA repair pathway
Source: Nat Commun. 2022 Sep 9;13:5303. doi: 10.1038/s41467-022-33027-z (PMC9463442; doi:10.1038/s41467-022-33027-z)
Supplement: Supplementary file 2 — Description of Additional Supplementary Files [file 41467_2022_33027_MOESM2_ESM.pdf]

Description of Additional Supplementary Files

File Name: Supplementary Movie 1
Description: Video shows CtIP-GFP protein recruitment 10 minutes after microirradiation. Left, representative DMSO-treated cells. Right, representative THZ1- treated cells during 2 hours 10 µM previous irradiation. N=3 independent experiments.
