# Supplementary material for: CtIP-dependent nascent RNA expression flanking DNA breaks guides the choice of DNA repair pathway
Source: Nat Commun. 2022 Sep 9;13:5303. doi: 10.1038/s41467-022-33027-z (PMC9463442; doi:10.1038/s41467-022-33027-z)
Supplement: Supplementary file 4 — Reporting Summary [file 41467_2022_33027_MOESM4_ESM.pdf]

nature research

Corresponding author(s): Jiri Bartek
Daniel Gómez-Cabello

Last updated by author(s): Aug 19, 2022

Reporting Summary

Nature Research wishes to improve the reproducibility of the work that we publish. This form provides structure for consistency and transparency in reporting. For further information on Nature Research policies, see Authors & Referees and the Editorial Policy Checklist.

Statistics

For all statistical analyses, confirm that the following items are present in the figure legend, table legend, main text, or Methods section.

n/a	Confirmed	
☐	☒	The exact sample size (*n*) for each experimental group/condition, given as a discrete number and unit of measurement
☐	☒	A statement on whether measurements were taken from distinct samples or whether the same sample was measured repeatedly
☐	☒	The statistical test(s) used AND whether they are one- or two-sided<br>*Only common tests should be described solely by name; describe more complex techniques in the Methods section.*
☒	☐	A description of all covariates tested
☐	☒	A description of any assumptions or corrections, such as tests of normality and adjustment for multiple comparisons
☐	☒	A full description of the statistical parameters including central tendency (e.g. means) or other basic estimates (e.g. regression coefficient) AND variation (e.g. standard deviation) or associated estimates of uncertainty (e.g. confidence intervals)
☒	☐	For null hypothesis testing, the test statistic (e.g. *F*, *t*, *r*) with confidence intervals, effect sizes, degrees of freedom and *P* value noted<br>*Give P values as exact values whenever suitable.*
☒	☐	For Bayesian analysis, information on the choice of priors and Markov chain Monte Carlo settings
☒	☐	For hierarchical and complex designs, identification of the appropriate level for tests and full reporting of outcomes
☒	☐	Estimates of effect sizes (e.g. Cohen's *d*, Pearson's *r*), indicating how they were calculated

Our web collection on statistics for biologists contains articles on many of the points above.

Software and code

Policy information about availability of computer code

Data collection	Zeiss ZEN software, for LSM microscope image acquisition<br>PerkinElmer UltraView VoX and images were taken using Nikon Eclipse Ti microscope, microlaser irradiation<br>scanR acquisition software 3.0, for images taken in Olympus high-content screening microscope<br>Image Lab software 5.1 (Bio-Rad) for western blot detection.
Data analysis	ZEN Blue Edition software, analysis of LSM-acquired micrographs<br>scanR analysis software 3.0, analysis of high-content microscope acquired images<br>Image J 1.52k, kinetics analysis and colocalization<br>GraphPad Prism 8, numerical and statistical analysis<br>Adobe Illustrator 2021, figure assemply<br>Fiji, version 1.52u

For manuscripts utilizing custom algorithms or software that are central to the research but not yet described in published literature, software must be made available to editors/reviewers. We strongly encourage code deposition in a community repository (e.g. GitHub). See the Nature Research guidelines for submitting code & software for further information.

Data

Policy information about availability of data

All manuscripts must include a data availability statement. This statement should provide the following information, where applicable:

- Accession codes, unique identifiers, or web links for publicly available datasets
- A list of figures that have associated raw data
- A description of any restrictions on data availability

All raw data associated to Figures in manuscript are provided as supplementary material and Source Data Excel file.

Field-specific reporting

Please select the one below that is the best fit for your research. If you are not sure, read the appropriate sections before making your selection.

☒ Life sciences ☐ Behavioural & social sciences ☐ Ecological, evolutionary & environmental sciences

For a reference copy of the document with all sections, see nature.com/documents/nr-reporting-summary-flat.pdf

Life sciences study design

All studies must disclose on these points even when the disclosure is negative.

Sample size	No statistical method was used to predetermine sample size. For DNA fiber experiments, if equivalent experiments were not different statistically (p-value of t-test <0.001), the total number of DNA fibers from all experiments is presented. Untreated control cells, with drug vehicle, or cells transfected with non-targeting siRNA (siNT) control were included as appropriate controls for each specific experiment.
Data exclusions	No exclusion was applied.
Replication	For all experiments, the number of biological replicates is indicated and reproduced the representative data are shown in figures.
Randomization	No human participants or animal models were reported in this manuscript. Samples were organized into different treatments (cell type/ chemical treatment/knockdown conditions).
Blinding	Investigators were blinded during DNA- and RNA fiber scoring and high-content image analysis and also in protein recrutiment kinetics. For the automated acquisition of DDR foci blinding was not necessary due to intrinsically unbiased nature of the approach.

Reporting for specific materials, systems and methods

We require information from authors about some types of materials, experimental systems and methods used in many studies. Here, indicate whether each material, system or method listed is relevant to your study. If you are not sure if a list item applies to your research, read the appropriate section before selecting a response.

Materials & experimental systems

n/a	Involved in the study
☐	☒ Antibodies
☐	☒ Eukaryotic cell lines
☒	☐ Palaeontology
☒	☐ Animals and other organisms
☒	☐ Human research participants
☒	☐ Clinical data

Methods

n/a	Involved in the study
☒	☐ ChIP-seq
☒	☐ Flow cytometry
☒	☐ MRI-based neuroimaging

Antibodies

Antibodies used

Brdu: APBiotech RPN20AB_RPN202 (1:500), anti-Rat
BrdU:BD Biosciences 347580 (1:500), anti-mouse.
S9.6: keraFAST ENH001 (1:250), anti-mouse.
53BP1: millipore MAB3802 (1:500), anti-mouse.
H2AX-S139: Millipore 16-193 (1:1000), anti-mouse.
H2AX-S139: Cell Signaling S2577s (1:500),anti-rabbit.
BRCA1: Santa Cruz sc-6954 (1:200), anti-mouse.
RNAPol II: Abcam ab817 (1:2000), anti-mouse.
S5P RNAPol II: Abcam ab5131 (1:500), anti-rabbit.
CtIP: active motif 61141 (1:500), anti-mouse.
RAD52:Santa Cruz sc-365341 (1:500), anti-mouse.
alpha-tubulin: Gene Tex GTX628802 (1:2000), anti-mouse.
S4/S8 RPA:Bethyl A300-245A (1:500), anti-rabbit.
RAD51:Abcam ab63801 (1:200), anti-mouse.
PCNA:immuno concepts 2037 (1:1000), anti-human.
RIF1: Bethyl A300-569A (1:500), anti-rabbit
ALEXA-Fluor 488 GOAT Invitrogen A11029 Mouse (1:1000), anti-mouse.
ALEXA-Fluor 568 GOAT Invitrogen A11036 Rabbit (1:1000), anti-rabbit.
ALEXA-Fluor 555 GOAT Invitrogen A21424 Mouse (1:1000), anti-mouse.
ALEXA-Fluor 647 GOAT Invitrogen A21445 Human (1:1000), anti-human.
ALEXA-Fluor 568 GOAT Invitrogen A11077 Rat (1:1000), anti-rat.

Validation	Rat anti-BrdU antibody, Validated by the company and cited in more than 159 research papers by immunofluorescence. We cited this antibody in Rasmussen et al. 2016. Nat Comm. 15 Nov.<br>BrdU (BD Biosciences, #347580), cited in 736 research papers by immunofluorescence.<br>H2AX-S139 (Millipore 16-193) , Quality assurance by the company for Immunoblot Analysis and Immunocytochemistry in human cells cited by 2303 research papers by immunofluorescence.<br>RAD51, Cited and validated in more than 30 research papers by immunofluorescence. We cited this antibody in Moudry et al. 2016. JCB 212:281-288.<br>RPA32 S4/8, Validated and cited in 126 research papers including human cells by immunofluorescence.<br>53BP1, Validated by the company and cited in 427 research papers by immunofluorescence and western blot. We cited this antibody in Burrel et al. 2013, Nature 494:492-6.<br>S9.6 (hybridoma cell line HB-8730) cited at least by 30 papers and validated in human cells by immunofluorescence.<br>The rest of antibodies were validated by the manufacturers. Methods of validation such as immunofluorescences and western blots and references have been published for all antibodies are all present into manufacturer dedicated website page of each indicated product.

Eukaryotic cell lines

Policy information about cell lines

Cell line source(s)	U2-OS (ATCC HTB-96)<br>HeLa (ATCC CCL-2)
Authentication	Cell lines were purchased from ATCC and authenticated by STR method.
Mycoplasma contamination	All cell lines used in this study were negative for mycoplasma. We regularly check by PCR the presence of mycoplasma in all cell lines used in our laboratory.
Commonly misidentified lines<br>(See ICLAC register)	No commonly misidentified cell lines were used in this study.
